# Nonspherical Particle Stabilized Emulsions Formed through Destabilization and Arrested Coalescence

**DOI:** 10.1021/acs.langmuir.4c03812

**Published:** 2024-12-26

**Authors:** Benjamin T. Lobel, Daniele Baiocco, Mohammed Al-Sharabi, Alexander F. Routh, Zhibing Zhang, Olivier J. Cayre

**Affiliations:** † School of Chemical and Process Engineering, University of Leeds, Leeds, LS2 9JT, United Kingdom; ‡ School of Chemical Engineering, University of Birmingham, Birmingham, B15 2TT, United Kingdom; § Department of Chemical Engineering and Biotechnology, University of Cambridge, Cambridge, CB3 0AS, United Kingdom

## Abstract

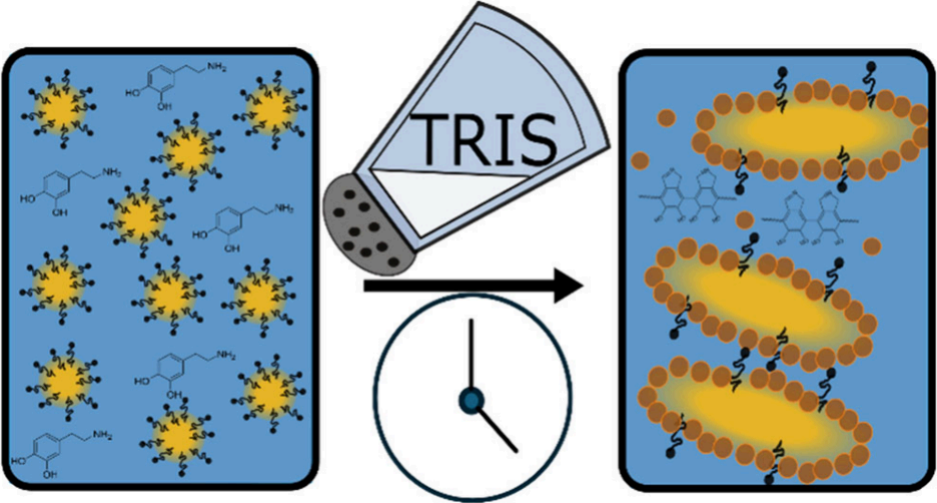

To form nonspherical emulsion droplets, the interfacial tension driving droplet sphericity must be overcome. This can be achieved through interfacial particle jamming; however, careful control of particle coverage is required. In this work, we present a scalable novel batch process to form nonspherical particle-stabilized emulsions. This is achieved by concurrently forming interfacially active particles and drastically accelerating emulsion destabilization through addition of electrolyte. To achieve this, surfactant-stabilized oil-in-water emulsions in the presence of dopamine were first produced. These emulsions were then treated with tris(hydroxymethyl)aminomethane hydrochloride buffer to both simultaneously initiate polymerization of dopamine in the emulsion continuous phase and reduce the Debye length of the system, thus accelerating droplet coalescence while forming surface-active particles. The concentration of buffer and imposed shear was then systematically varied, and the behavior at the interface was studied using pendent drop tensiometry and interfacial shear rheology. It was found that polydopamine nanoparticles formed in the emulsion continuous phase adsorbed to the reducing interface during coalescence, resulting in anisotropic droplets formed via arrested coalescence. Greater shear rates resulted in accelerated coalescence and formation of secondary droplets, whereas lower shear rates resulted in thicker interfacial films. The efficacy of this method was further demonstrated with a second system consisting of sodium dodecyl sulfate as the surfactant and polypyrrole particles, which also resulted in nonspherical droplets for optimized conditions.

## Introduction

Nonspherical or anisotropic emulsions may be beneficial in a number of applications owing to their increased surface area to volume ratio, including in catalysis, pharmaceuticals, nutrition and as templates for microencapsulation.^1−6^ This increase in surface area may result in increased target-adsorption efficiency and a reduction in material requirements to achieve desired effects.^7−10^ Specifically, this increased adhesion could be used to improve the efficiency of the deposition of capsules used in laundry and agricultural applications. In order to achieve nonspherical emulsions, the surface tension, driving both the reduction in interfacial area and consequently the droplet sphericity, must be overcome. This has previously been achieved in the contemporary literature not only for emulsion droplets but also for particles. For example, Roh et al. produced soft dendritic particles that demonstrated high adhesion to glass substrates.^11^ However, this method required careful tuning of solvent–polymer interactions and shear flow conditions. Likewise, Procter & Gamble have patented technology utilizing the internal freezing of an oil structure via in situ crystallization under shear, resulting in anisotropic surfactant-stabilized emulsions developed for enhanced substrate adhesion and active delivery.^12^ In this case, the mechanical attributes imparted by the crystallized wax resist the restoring force of surface tension. Recently, Lian et al. have produced nonspherical emulsions via coaxial flow and fast interfacial polymerization of butylcyanoacrylate resulting in ellipsoidal droplets.^13^ Notably, nonspherical droplets have also been formed by carefully controlled freezing of surfactant stabilized oil droplets.^14,15^ Nonspherical emulsion droplets have also been formed using particle stabilized emulsions. These emulsions differ from traditional surfactant emulsions as their primary stabilization is typically driven by steric interactions, resulting from irreversible particle adsorption on the droplet surface. The energy required to desorb a particle from the interface (Δ*E*) is a function of the particle radius (*R*), the contact angle (θ), and the bare aqueous/oil interfacial tension (*γ*_ow_)^16,17^ and can be calculated as follows:
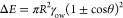
1where desorption energy is minimized at very low or high values of θ, i.e., where the particle is easily desorbed from the interface into the aqueous or oil phase. However, when the energy of desorption is high, the particles can be considered as irreversibly adsorbed. If particle stabilized emulsions are partially covered and begin to coalesce, they may undergo arrested coalescence, a process by which there is insufficient surface area on the newly formed (larger) droplet to accommodate all of the adsorbed particles, thus causing interfacial jamming. This jamming results in a mechanical resistance to completion of the coalescence process, driving nonspherical droplet shapes.^18−23^

A benefit of producing anisotropic particle-stabilized emulsions is the relative ease with which they may be further developed into microcapsules, providing protection of active ingredients not available to a simple emulsion system.^6^ Bon and co-workers produced nonspherical droplets by forcing laponite-stabilized droplet coalescence through a narrow capillary, while Subramaniam et al. reported the formation of anisotropic particle-stabilized bubbles by pressing partially coated bubbles together between two glass slides.^22,23^ Another reported method for forming nonequilibrium shapes in particle stabilized emulsions is via nanoparticle surfactants. These systems are composed of particles made surface active via electrostatically driven interfacial interactions, based on interactions with oppositely charged species in the opposite phase. Such systems have been shown to form nonspherical emulsions and simple bijels; biphasic continuous liquid/liquid systems stabilized by jammed interfacially active particles but typically require carefully chosen polymer, particle, and solvent combinations.^24,25^ Although effective, these aforementioned methods require the use of specialist equipment and specific material interactions or are only capable of producing droplets sequentially, which may result in challenging scale-up.

Herein we report the formation of nonspherical emulsions stabilized by a combination of surfactants and polymer nanoparticles via a simple and potentially scalable process with no requirement for bespoke or specialist equipment or techniques. These emulsions are formed via in situ polymerization during electrolyte induced emulsion destabilization/coalescence. By exploiting the coalescence of a destabilizing emulsion and the kinetics of a chemical oxidative polymerization process, the interface of coalescing droplets is intentionally jammed, resulting in anisotropic particle-stabilized emulsions. In developing these systems, we investigate the impact of the destabilizing electrolyte concentration and the shear rate during the coalescence/polymerization processes. Furthermore, we show that anisotropic emulsion droplets can also be obtained with this method by using a second system. Thus, we demonstrate the scalable formation of anisotropic droplets with significantly different physicochemical properties and show that this mechanism may be applied to various systems.

## Experimental Section

### Preparation of Anisotropic Emulsions

**Figure 1:**
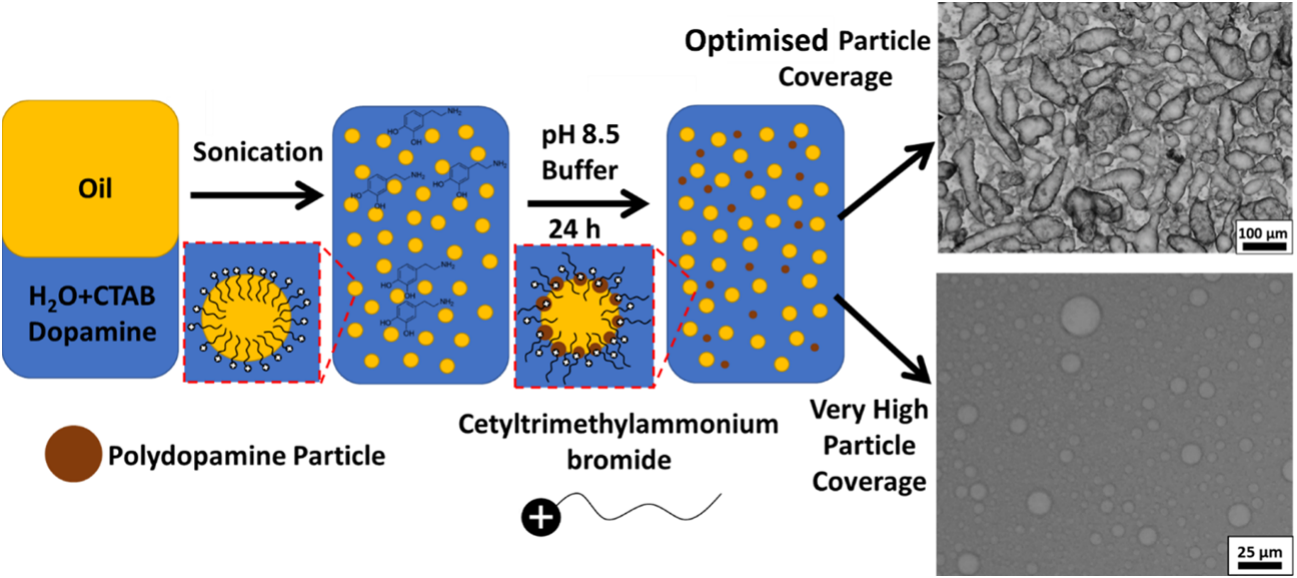
Schematic of anisotropic emulsion formation. Initial CTAB-stabilized oil/water emulsion formed with dopamine monomer present in the aqueous continuous phase. pH 8.5 buffer was added while stirring, thus inducing chemical oxidative polymerization of dopamine and simultaneous electrostatically driven emulsion destabilization (coalescence). Polydopamine particles are formed in the continuous phase and stabilize the interface, while some oligomers also remain in the continuous phase, resulting in nonspherical emulsion droplets (right). The top micrograph was produced by using a concentration of 0.032 M dopamine hydrochloride. The bottom micrograph was produced using a concentration of 0.10 M dopamine hydrochloride. Both emulsions were produced by using 0.34 M Tris-HCl and 0.38 mM CTAB.

Unless otherwise stated, all materials were used as received. 3.0 mL of a dopamine hydrochloride solution (0.05 M, Alfa Aeser, United States) was placed in a 20 mL vial followed by 100 μL of 18 mM cetyltrimethylammonium bromide (CTAB, Panreac AppliChem, United States) and mixed to form the aqueous phase. 250 mg of hexadecane (Sigma-Aldrich, UK) was then added (oil phase), gently shaken by hand to mix the two phases, and then emulsified using an ultrasonic probe (Fischer FB505, 20 kHz, 500 W) for 1 min at 20% amplitude while the vial was immersed in a water bath at room temperature (RT, 22 °C) to ensure constant temperature. A magnetic stir bar was added to the newly formed emulsion, and the mixture was allowed to stir for 30 min. Following this, 1.6 mL of tris(hydroxymethyl)aminomethane hydrochloride (Tris-HCl buffer, pH 8.5, Sigma-Aldrich, UK) was added to the emulsion to both initiate polymerization and accelerate coalescence. The emulsion was allowed to stir for 24 h before being centrifuged (Megafuge 16R, Thermo Scientific) at 2000 RCF for 20 min to separate emulsion droplets from free polydopamine (PDA) particles dispersed in the continuous phase (Figure 1, PDA/CTAB emulsion). Preliminary experiments varying monomer concentration were also performed with results being presented in Supporting Information (Figure S1)

In order to demonstrate that this arrested coalescence mechanism, leading to the production of nonspherical droplets, was not exclusive to the dopamine/CTAB system, a second emulsion system was tested where the monomer pyrrole (Py, Sigma-Aldrich, UK) was used instead of dopamine. 3.0 mL of 1 mM solution of sodium dodecyl sulfate (SDS, Sigma) was combined with 250 mg of hexadecane and subjected to emulsification as outlined above. 160 mg of Py, which was purified via a basic alumina column (Brockman’s I, Sigma-Aldrich, UK) before addition, was added to the emulsion and allowed to stir for 1 h. Finally, 1.6 mL of 150 mM H_2_PtCl_6_.*x*H_2_O (Sigma-Aldrich, UK) was then added to the emulsion before leaving the system to polymerize and coalesce while stirring for 7 days (Figure S2) based on previous work by Takeoka et al.^26^ Particles formed in the bulk were separated at the end of this process via centrifugation, as outlined above. The resulting emulsions henceforth referred to as PPy-Pt/SDS

### Optical, Fluorescent and Cryogenic Scanning Electron Microscopy

Optical micrographs were obtained by using an Olympus BX51 microscope with a fluorescent light source (Olympus U-LH100HG). Hexadecane spiked with Nile Red was used to monitor the presence of the oil throughout the process, which verified that no significant phase separation occurred. Confocal fluorescence images of the PPy-Pt/SDS emulsions were captured on a Zeiss LSM880 microscope. Cryogenic SEM samples were prepared using a freezing rivet that was submerged in liquid nitrogen before being transferred under a vacuum into a Quorum PP3010 cryo-preparation chamber. The sample was fractured using a cooled knife and allowed to sublime to reveal emulsion droplets beneath the ice layer. An iridium coating was sputtered onto the samples before being transferred into a Thermo scientific Helios G4 CX DualBeam operating at an accelerating voltage of 2 kV. Cryo conditions were maintained throughout analysis.

### Impact of Electrolyte Concentration

The effect of electrolyte concentration on the resultant emulsion shape was explored by varying the concentration of the Tris-HCl buffer (from 0 to 0.64 M) added to the emulsion while maintaining a constant volume and a pH of 8.5.

The emulsion droplet shape was evaluated by using a FlowCam cytometer (C70 Benchtop, FluidImaging). The FlowCam was programmed to take images until 1 million droplets were identified by the software in AutoImage mode using a 10× objective for most emulsions and 20× for the 0 M Tris-HCl sample due to its smaller size. A FC100 × 2 flow cell was used in a reverse flow configuration for these measurements due to the density difference between the oil and continuous phase. Post capture, the droplet images were processed with VisualSpreadsheet native instrument software, eliminating droplets out of focus using the edge gradient function. The anisotropy of the droplets was assessed using the circle fit software function (further details in SI).

### Impact of Shear Rate

To investigate the impact of shear rate on the shape and characteristics of the emulsions, additional emulsions were formed as outlined above, with all concentrations kept constant. Volumes were increased by a factor of 11 to facilitate sufficient submersion of the impeller. Emulsions were also formed via sonication for 11 min, instead of 1 min, in a 100 mL round bottomed flask to maintain a constant J/m^3^ energy input during emulsification. This flask was then subjected to shear by using an overhead stirrer with an impeller (IKA, blade length 3.8 cm) for 24 h at 250, 375, and 500 rpm instead of using a magnetic stirrer. While RPM is not the same as shear rate, the shear rate for a given geometry and viscosity is proportional to the impeller RPM, and thus by increasing impeller RPM, the shear rate is also increased. Samples were kept in a 25 °C water bath during sonication and polymerization. Difference in size distribution between these samples was investigated using laser diffraction (Malvern Mastersizer 3000) equipped with a hydrodispersion unit (Hydro-MV) and sphericity measured using the Flowcam as outlined above.

### Zeta Potential

An aqueous phase was prepared as outlined above but without the addition of an oil phase or use of the ultrasonic probe, resulting in a PDA particle dispersion (PDA-AP). Briefly, a 3.1 mL solution containing 0.15 mmol of dopamine hydrochloride and 1.8 μmol of CTAB was combined with 1.6 mL of Tris-HCl buffer (pH 8.5, 1 M) in a 20 mL vial and allowed to polymerize for 24 h under magnetic stirring (Figure S3). Following this, a dialyzed sample was prepared by placing the PDA dispersion in dialysis tubing (MWCO 8000 Da) and placed in a 2.5 L beaker of water. Water was then changed daily until conductivity and pH were the same as that of pure water, thereby removing excess surfactant, buffer, monomer, and oligomer <8 kDa (PDA-D). This sample was then used as the aqueous phase, before separate addition of Tris-HCl buffer (PDA-Tris) and CTAB (PDA-CTAB) and a combination of both (PDA-Tris/CTAB) to match the conditions used in initial particle/emulsion preparation. The zeta potential of the PDA particle systems was measured using a Malvern Zetasizer Ultra using a DTS1070 Malvern cell.

### Interfacial Tension and Shear Rheology

Interfacial tension (IFT) for each particle dispersion prepared was measured against a hexadecane oil phase. This was achieved using the pendent drop method.^27,28^ The bare water/hexadecane interface was measured prior to any additional measurements to ensure the correct fitting and to establish a baseline. In each case, the aqueous phase was injected into the oil phase until the characteristic pendent droplet was able to form due to the force balance between interfacial and gravitational forces. The Young–Laplace equation was then fitted to the droplet by the native instrument software to determine *γ*_ow_.^27,29^ Videos are presented in Supporting Information (Videos S1–3).

Interfacial shear rheology (IFSR) was also performed using PDA-AP and PDA-Tris/CTAB using a hexadecane oil phase for comparison (TA Discovery HR-2, with double wall ring geometry). The base receptacle was first filled with 19.3 mL of the aqueous phase, and the ring was lowered to be in contact with the liquid. The ring was then lowered until it was half covered, and hexadecane was added to the receptacle to form an o/w interface. Initial studies were performed to measure the elastic/storage (*G*′) and viscous/loss modulus (*G*″) at a 1% strain and 0.5 Hz oscillation rate for 1 h. Following this, the samples were subjected to a strain amplitude sweep from 0.01 to 100% to investigate the yield strain of the formed films.

## Results and Discussion

### Preparation of Anisotropic Emulsions

**Figure 2:**
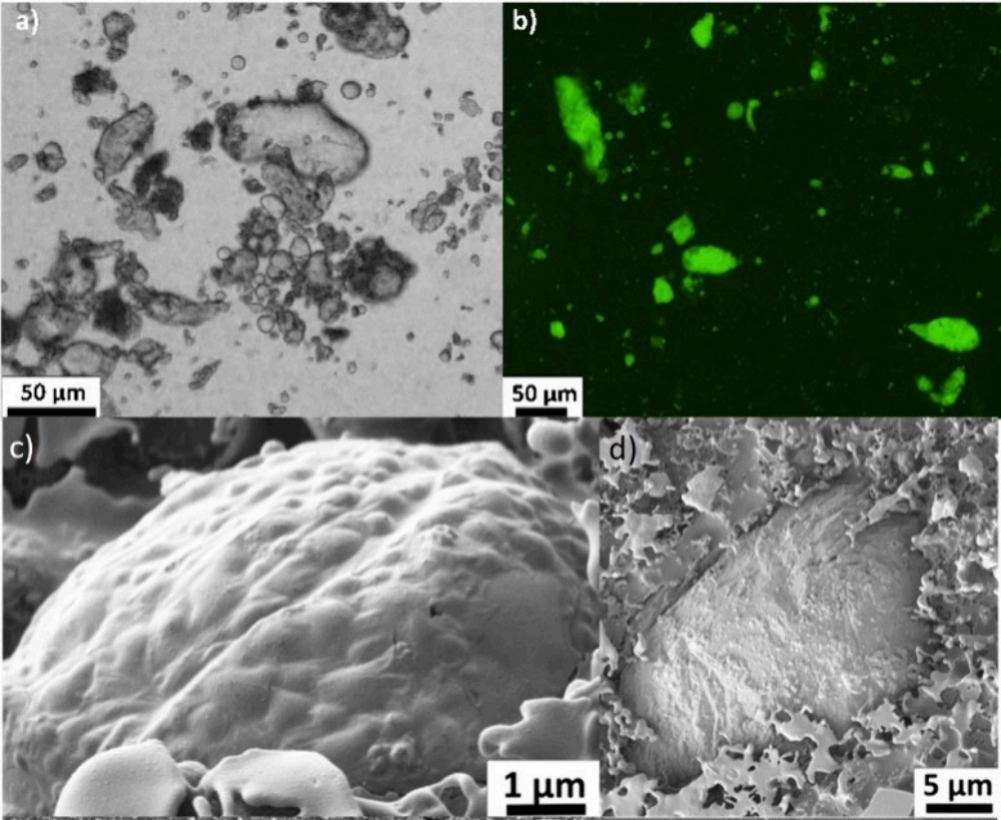
Micrographs of PDA/CTAB nonspherical emulsions. a) Optical micrograph of emulsion formed with electrolyte concentration of 0.32 M. b) Repeated emulsion stained with Nile Red under fluorescence obtained using optical microscopy. (c,d) CryoSEM micrographs of emulsion droplets for a sample prepared using 0.32 M Tris-HCl and 0.38 mM CTAB.

Micrographs reveal the presence of a large number of nonspherical droplets in the PDA/CTAB emulsion (Figures 1 and 2). Furthermore, the use of CryoSEM allowed for a visual inspection of the surface of the emulsion droplets. These emulsion droplets appear to be armored with particles of various sizes and exhibit a high aspect ratio ellipsoid morphology, which is characteristic of an arrested coalescence phenomenon.^18−21,30^ In this case, the primary emulsion is stabilized by cationic surfactants. The repulsion is based on electrostatic interactions provided by the surfactant, which generates this stabilization and is governed by classic DLVO theory.^31,32^ Upon the addition of relatively concentrated electrolyte (Tris-HCl), the reduction in Debye length initiates an increased coalescence rate. Simultaneously, the electrolyte results in the chemical oxidative polymerization of dopamine to polydopamine (PDA), forming PDA particles in the continuous phase.^33,34^ These polymer particles may then electrostatically interact with the surfactant, increasing their surface activity and driving increased droplet surface coverage with time. Specifically, the negatively charged catechol group present in PDA will interact with the cationic quaternary ammonium headgroup of CTAB.^35^. This complementary particle/surfactant interaction is reported throughout the literature for a number of particle/surfactant combinations.^36−40^ As a result of these interactions, the particles are driven to the oil–water interface and contribute to the stabilization of the corresponding emulsion droplets.

**Figure 3:**
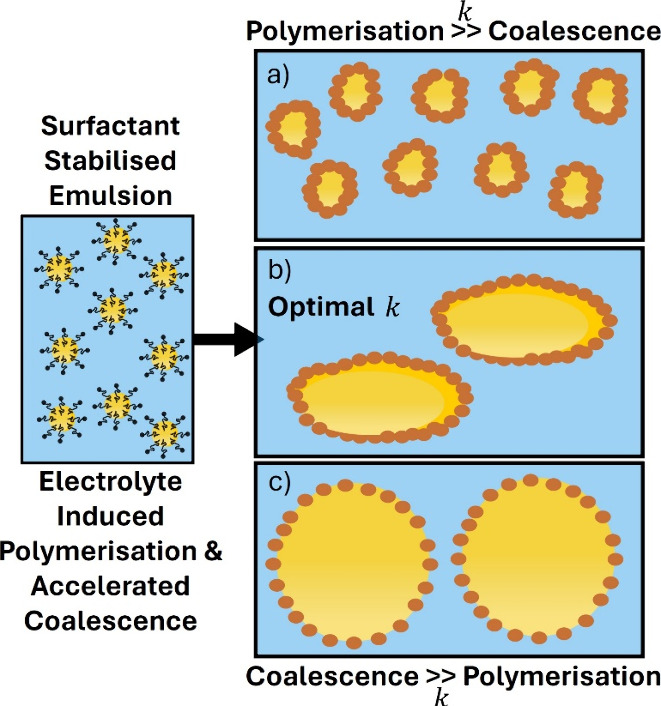
Schematic of salt induced coalescence and polymerization from primary emulsion, based on kinetics (*k*) a) Polymerization is fast relative to coalescence and particles stabilize small droplets. b) Similar kinetics of both processes, leading to arrested coalescence as particles adsorb to the reducing interface. c) Coalescence is fast relative to polymerization leading to large coalesced, primarily spherical droplets stabilized by polymer particles (Figure S1).

In summary, while the interfacial area of the emulsion decreases with time due to coalescence, the concentration of the effective emulsifier present in the system increases. Consequently, as the stabilizing PDA particles are irreversibly adsorbed at the interface^16,41,42^ (eq 1), the particles become jammed at the interface and resist the interfacial tension driving the coarsening and reduction in sphericity of the droplets. This results in observed morphological anisotropy. Indeed, this process is a kinetic balance between the electrolyte-induced coalescence of the surfactant stabilized emulsion and the formation and adsorption of the polymer particles produced in situ. In addition, an optimized ratio between the O/W interfacial area present in the system and the number of particles formed exist. If this ratio is too low (i.e., a higher concentration of dopamine is used), droplets are too efficiently stabilized by the formed polydopamine particles and do not undergo the arrested coalescence process. Pawar et al. reported that for a two-droplet coalescence event, when combined fractional particle interfacial coverage was above 0.9, no coalescence was possible, and below 0.7 complete coalescence occurred, and only between these two coverages could arrested coalescence take place.^19^ This built upon the previous work of Golemanov et al, who reported arrested coalescence as a function of droplet diameter and particle concentration.^20^ Thus, if coalescence is slow relative to particle nucleation, more particles are able to stabilize the droplets and coalescence is subsequently inhibited. Conversely, if coalescence is too fast then insufficient coverage occurs, resulting in complete coalescence (Figure 3). Thus, for comparison, we separately monitored the kinetics of both PDA polymerization and the destabilization of the CTAB emulsions (Supporting Information, Figures S4–S8). Polymerization kinetics were followed by ^1^HNMR spectroscopy using the disappearance of the dopamine monomer peak over time.^44^ These results showed that over 20% of the monomer is consumed 2 h after addition of the Tris buffer, which starts the polymerization reaction. After 8h, 60% of the monomer was used in the polymerization, which appeared to be complete after 24h (with no monomer detected in the system by that point, see Figures S4–S7). These data can be compared to those obtained when monitoring the emulsion droplet size increase over time, which is indicative of the coalescence kinetics (Figure S8). On similar time scales, between 3 and 5 h after buffer addition, the emulsion droplet size distribution begins to broaden and shifts to larger sizes. This is an important observation as if the polymerization and coalescence kinetics were not comparable (i.e., polymerization or coalescence was too fast/slow), then arrested coalescence would not occur. Specifically, if droplets completely coalesce before particle formation, then only large spherical droplets would result from this process. Conversely, if particles fully form before droplet coalescence occurs, the process will lead to small spherical particle-stabilized emulsions (Figures 1 and 3).

CryoSEM images (Figures 2c,d) and associated EDX elemental mapping (Figure S9) indicate that the droplets are covered with a film, in which discrete particles appear to be embedded. This is concordant with the proposed formation mechanism of initial particle stabilized emulsion formation reinforced by a polymeric film. In addition, the CryoSEM images demonstrate that despite the nonsphericity being driven by coalescence of smaller droplets, resulting in large droplets (length >50 μm), smaller (length of ∼10 μm) nonspherical droplets are also still present in the sample, likely as a result of small droplets with a high polydopamine particle coverage coalescing together into an arrested shape.

### Impact of Electrolyte

**Figure 4:**
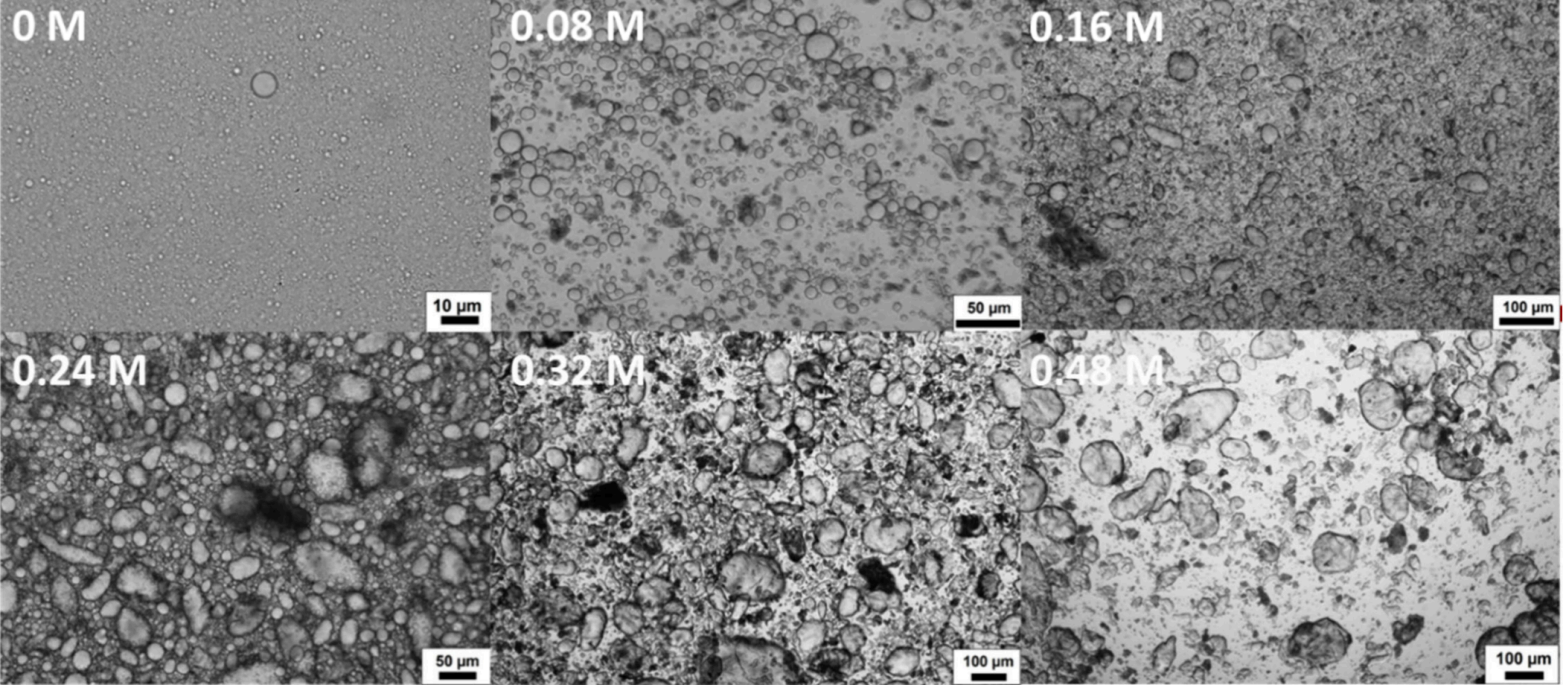
Micrographs of PDA/CTAB emulsion formed by increasing the final electrolyte concentration. Concentrations in each image correspond to final Tris-HCl concentration during coalescence. All emulsions were initially formed using 0.38 mM CTAB.

The primary emulsion is electrostatically stabilized by the cationic surfactant CTAB (0.38 mM). This emulsion consists of droplets of approximately 1 μm diameter and is stable to coalescence and creaming (Figure 4 and Figure S8). Upon addition of electrolyte, which initiated the polymerization and accelerated coalescence, the droplets start increasing in size and become less spherical. The extent of this coalescence and droplet anisotropy is proportional to the concentration of electrolyte in the aqueous phase and the consequent extent of screening of the electrostatic-driven repulsion generated by the CTAB adsorbed on the droplet surfaces (Figure 4). It is not expected that the increase in buffer concentration will substantially affect the polymerization kinetics, which is primarily first order with respect to dopamine monomer concentration.^45^ In addition to the increase in size and anisotropy, the droplets appear to become covered by an interfacial film as the electrolyte concentration increases, which becomes visibly coarser both over time and proportionally to the electrolyte concentration. The presence of this film is apparent when comparing the samples prepared with 0.08 and 0.48 M Tris-HCl concentrations. These findings are comparable to those reported by Tyowua et al., who produced nonspherical emulsions by preparing Pickering emulsions stabilized by CaCO_3_ in varying NaCl concentrations.^46^ They proposed that electrostatic stabilization was achieved by interparticle repulsion between the adsorbed CaCO_3_ particles (diameter between 1–7 μm) and only occurred at certain salt concentration ranges_._ However, the method reported herein relies on in situ generation of the particulate stabilizer and a synergistic effect of the forming particles and surfactant at the oil/water interface during coalescence. Furthermore, here the initial emulsion droplets are small (<1 μm), as a result of the initial emulsion being stabilized by a surfactant, instead of large particles, thus resulting in smaller micron-sized nonspherical emulsion droplets at the end of the polymerization/coalescence process.

**Figure 5:**
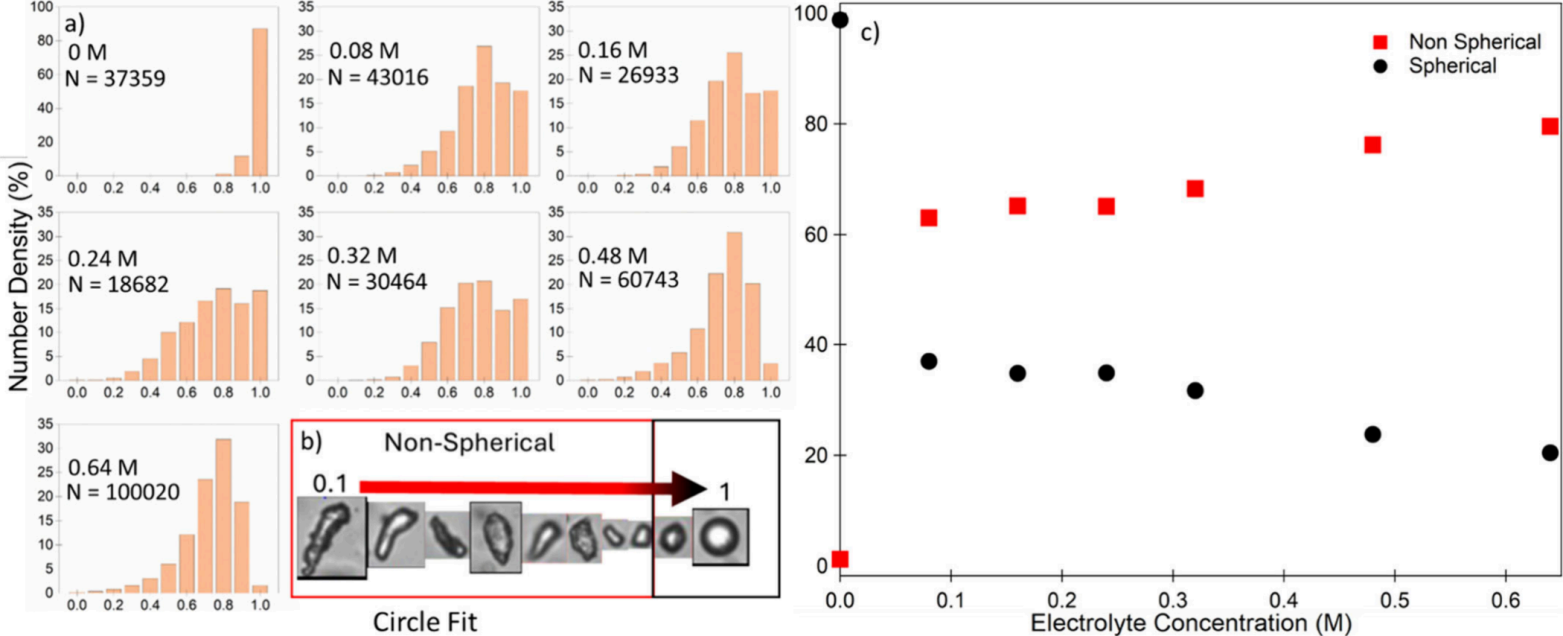
Number density of spherical and nonspherical droplets as a function of increasing electrolyte concentration during emulsion preparation for PDA/CTAB emulsions. a) Full distributions of circle fit measurements performed using FlowCam and number of droplets measured (N). The 0 M sample represents a sample that is not destabilized and was used to classify droplets as being spherical or nonspherical. b) Examples of droplets of increasing circularity and examples of image quality obtained from the FlowCam analysis of droplets for each electrolyte concentration including at least 2 repeats of each condition. Droplets are classified as being spherical if the circle fit was ≥0.85. c) Cumulative number density of particles being classified as spherical or nonspherical as outlined above as a function of electrolyte concentration.

Emulsion droplet characterization (using a Flowcam instrument, Figure 5) confirmed the qualitative observations made above for emulsions processed with increasing Tris-HCl concentration. The circle fit function was chosen to quantify the emulsion anisotropy. This measures the deviation of the captured droplet profile from that of a projected circle (circle fit = 1). The inset in Figure 5 presents examples of droplets of increasing circle fit from 0.1 to 1, where 1 represents a perfect sphere, and 0.1 is the shape deemed furthest away from sphericity. Based on these images, and the circle fit distribution of the CTAB-stabilized emulsion (Figure 5), droplets were characterized as either being spherical (circle fit ≥0.85) or nonspherical (circle fit ≤0.84). As expected, when there was no added Tris-HCl, the emulsion was almost completely spherical. However, on addition of electrolyte, this circle fit decreased as the emulsion evolved from surfactant-stabilization to a system where the oil–water interface is partially stabilized by both particles and surfactant while undergoing coalescence. When an increasing concentration of Tris-HCl was used to polymerize and destabilize the emulsion, the degree of anisotropy slowly increased. This reached a maximum within the range tested at 78% of droplets being nonspherical (Figure 5).

This is concordant with the proposed mechanism of an arrested coalescence phenomenon. As the emulsion coalesces, the droplets become increasingly larger, and as outlined above, the total interfacial area decreases until it can no longer support the number of polymeric particles adsorbed at the interface. As a result, the larger droplets are more likely to result in arrested coalescence.^18−21,46,47^

### Impact of Shear Rate

**Figure 6:**
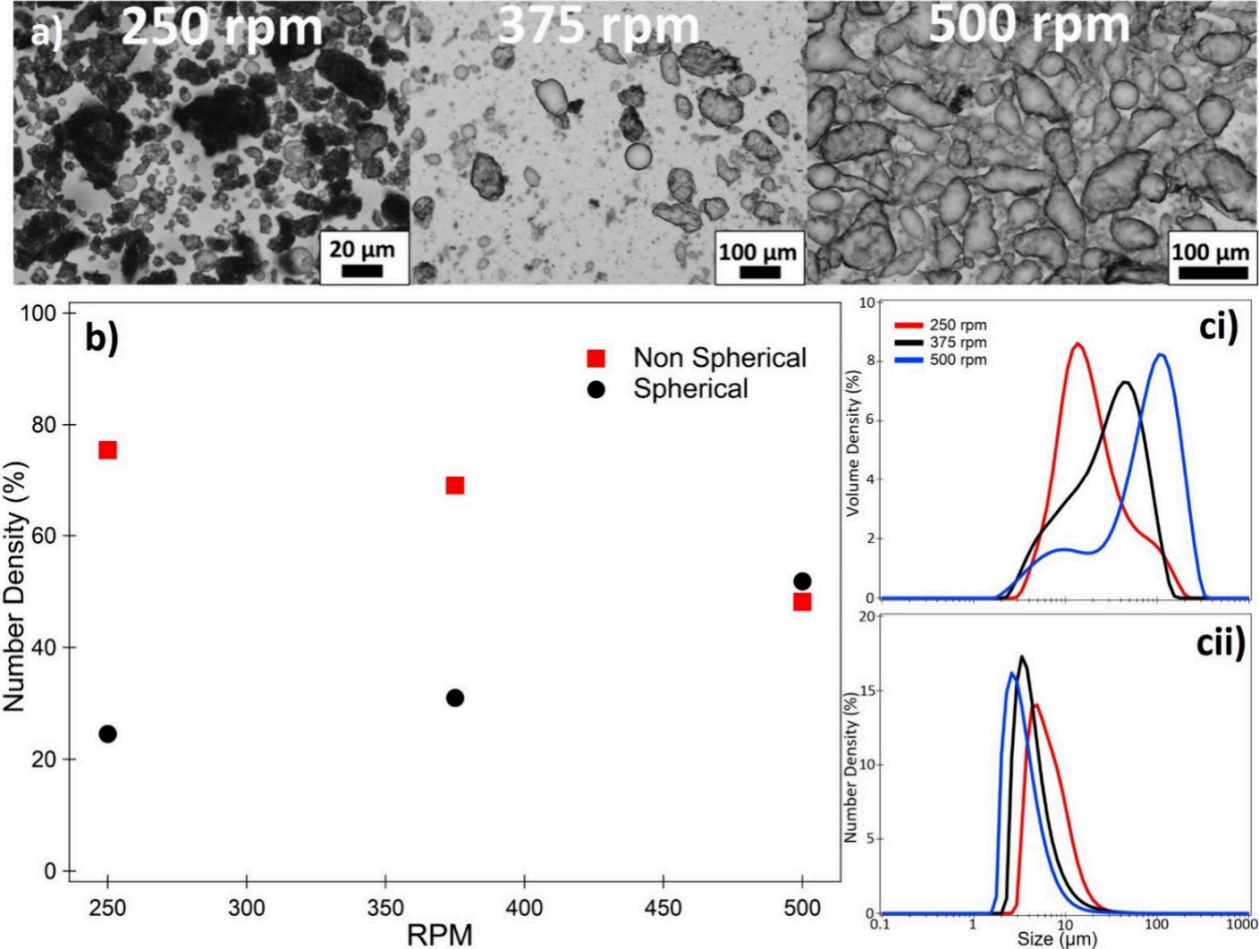
Impact of stirrer rotation speed (shear rate) during emulsion preparation on the size and anisotropy of PDA/CTAB formed emulsion droplets at a Tris-HCl concentration of 0.32 M. (a) Optical micrographs at increasing shear rate. (b) Number percentage of spherical and nonspherical droplets as outlined in Figure 5 as a function of increasing shear rate. (ci) Laser diffraction determined size distribution (volume %) and number % (cii) as a function of shear rate. Droplets are classified as being spherical if the circle fit was ≥0.85 as demonstrated in Figure 5.

By using an overhead impeller, it was possible to maintain the same geometry while increasing the shear applied during the coalescence and polymerization process. Optical micrographs of the emulsions at increasing impeller rotation speed (Figure 6a) reveal that, particles appear to form a thicker PDA interfacial film at lower shear, resulting in opaque droplets. As the shear rate increases (375 rpm), the droplets exhibit thinner particle shells and there appears to be a larger number of smaller droplets or particles dispersed in the continuous phase. When the sample prepared at the highest shear tested (500 rpm), highly deformed emulsion droplets with an apparently thinner PDA interfacial film can be observed. On measurement of the droplet morphology using the FlowCam (Figure 6b), the increase in impeller RPM appears to result in an increased number of spherical droplets. However, for the same Tris-HCl concentration as the data presented in Figure 5, the reverse trend is seen with the overall number of nonspherical droplets being higher at low RPM.

The use of an overhead impeller not only ensures more uniform mixing compared to a magnetic stirrer but also results in an increased overall shear stress applied to the emulsion system. As a result, the increase in initial droplet anisotropy observed in Figure 6b when compared to Figure 5 can be explained via deformation of the emulsion droplets due to the applied shear field.^48,49^ This may seem counterintuitive, as at increasing RPM the droplets tend to become more spherical (by number), contradicting the micrographs. However, as the shear rate increases, two competing phenomena occur simultaneously. The shear accelerates coalescence in the droplets resulting in a shift of the volume distribution to larger diameters presented in Figure 6ci.^50^ However, on examination of the number distribution (Figure 6cii), the size distributions begin to shift to a smaller diameter when impeller RPM is increased, resulting in an increase in D[4,3], D[3,2] (albeit at a lower rate), and Dv50, while Dn50 decreases (Table 1). This is due to the high shear stress not only deforming the emulsion droplets but also further breaking them into smaller secondary droplets.^49^ These droplets, having not undergone the same initial coalescence/polymerization process become quickly stabilized by not only particles in the bulk but also any remaining excess surfactant in the continuous phase, and as a result, remain spherical. That is, they are not becoming partially coated and coalescing, leading to interfacial jamming, but rather becoming completely armored by the particles and surfactant. These results can be related to work carried out by Whitby et al., who reported that at sufficiently high shear, not only do Pickering emulsion undergo coalescence, but that particles adsorbed at the interface may also become dislodged by the shear and return to the continuous phase.^50^ In such a case, these particles would then be available to stabilize the aforementioned secondary droplets, resulting in the increased occurrence of spherical droplets, or particle flocs. These simultaneous processes are concordant with the findings in Figure 6, where the detachment of particles from the interface coupled with droplet deformation at high shear result in high aspect ratio droplets possessing thinner films, while the presence of such particles and droplets decreases the median number diameter while simultaneously increasing the volume average diameter and sphericity.

**Table 1:** Droplet Diameters of PDA/CTAB Anisotropic Emulsions Prepared at Increasing Stirring Rates during Coalescence and Polymerization Determined by Laser Diffraction^a^

**Stirring Rate (RPM)**	**D[4,3]**^b^ **(μm) ± 3%**	**D[3,2]**^c^ **(μm) ± 3%**	**Dn50**^d^ **(μm) ± 3%**	**Dv50**^e^ **(μm) ± 3%**
250	27	18	5	24
375	34	22	6	31
500	97	32	3	88

a It is important to note that due to the nature of the Mastersizer measurements (assumption of spheroid particles) the exact numbers presented may not be reflective of the true particle size. However, the general trends and shifts of the distribution are still valid.

b D[4,3] De Brouckere or volume weighted mean diameter = .

c D[3,2] Sauter or mean equivalent sphere diameter of same volume/surface area =  where *n*_*i*_ and *d*_*i*_ are the number of particles of a given diameter and the diameter respectively.

d Dn50–Number weighted median diameter.

e Dv50–Volume weighted median diameter.

### Zeta Potential

PDA particles were separately prepared in an aqueous phase (in the absence of an oil phase) and the system was dialyzed to remove excess monomer, oligomers, and other contaminants. The zeta potential of this PDA particle suspension was then measured in pure water as well as in Tris-HCl buffer and after CTAB surfactant were added to the dispersion (Table 2). Interestingly, despite monomer not being present after 24 h of polymerization (Figures S7 and S9), the dialysis medium increased in opacity over time, and nanoparticles could be measured using dynamic light scattering (Figure S10).

**Table 2:** Zeta Potentials of PDA Particles before and after Dialysis and Addition of Surfactant and Buffer at Identical Concentrations to the Emulsions

**Sample**	**pH**	**Zeta Potential (mV)**
PDA-AP	8.5	–30 ± 1
PDA-D	6.7	–19 ± 0.4
PDA-CTAB	6.2	+33 ± 2
PDA-TRIS	8.6	–38 ± 0.2
PDA-CTAB/TRIS	8.6	+39 ± 1

The polydopamine particles prepared under the same conditions as the emulsions (without oil, PDA-AP) presented with a negative potential at the threshold of dispersion stability (−30 mV). This is in contrast to the dialyzed sample (PDA-D), which has a slightly less negative potential due to the change in pH between water (pH = 6.7), and Tris-HCl buffer (pH = 8.6). However, once dialyzed and the same amount of Tris-HCl buffer is re-added to the suspension (PDA-TRIS), the zeta potential becomes significantly more negative. While the increased negative charge when compared to PDA-AP is due to the presence of the CTAB in the PDA-AP and thus its particle charge is diminished through charge-screening. Conversely, the samples where CTAB were added show a positive zeta potential, demonstrating strong interaction between the surfactant and PDA particle surface, which initially is negatively charged. This change in polarity is characteristic of a bilayer of CTAB molecules assembling on the particle surface.^37,51^ Initially the cationic headgroup interacts with the anionic polymer particle, resulting in a net reduction in particle charge, however on further addition of CTAB, tail–tail interactions result in the slipping plane becoming positive.^37,51^ It is possible that, in the PDA-AP sample, the surfactant is electrostatically interacting with dissolved monomer or oligomer in the bulk and is incorporated into the particle rather than adsorbed at the surface, resulting in the negative zeta potential. Previous studies have reported that the presence of ionic surfactants can impact the polymerization and affect PDA film deposition.^52,53^ These oligomer-surfactant interactions appear to have significant implications on the film formation and are further investigated below.

### Interfacial Tension and Shear Rheology

**Figure 7:**
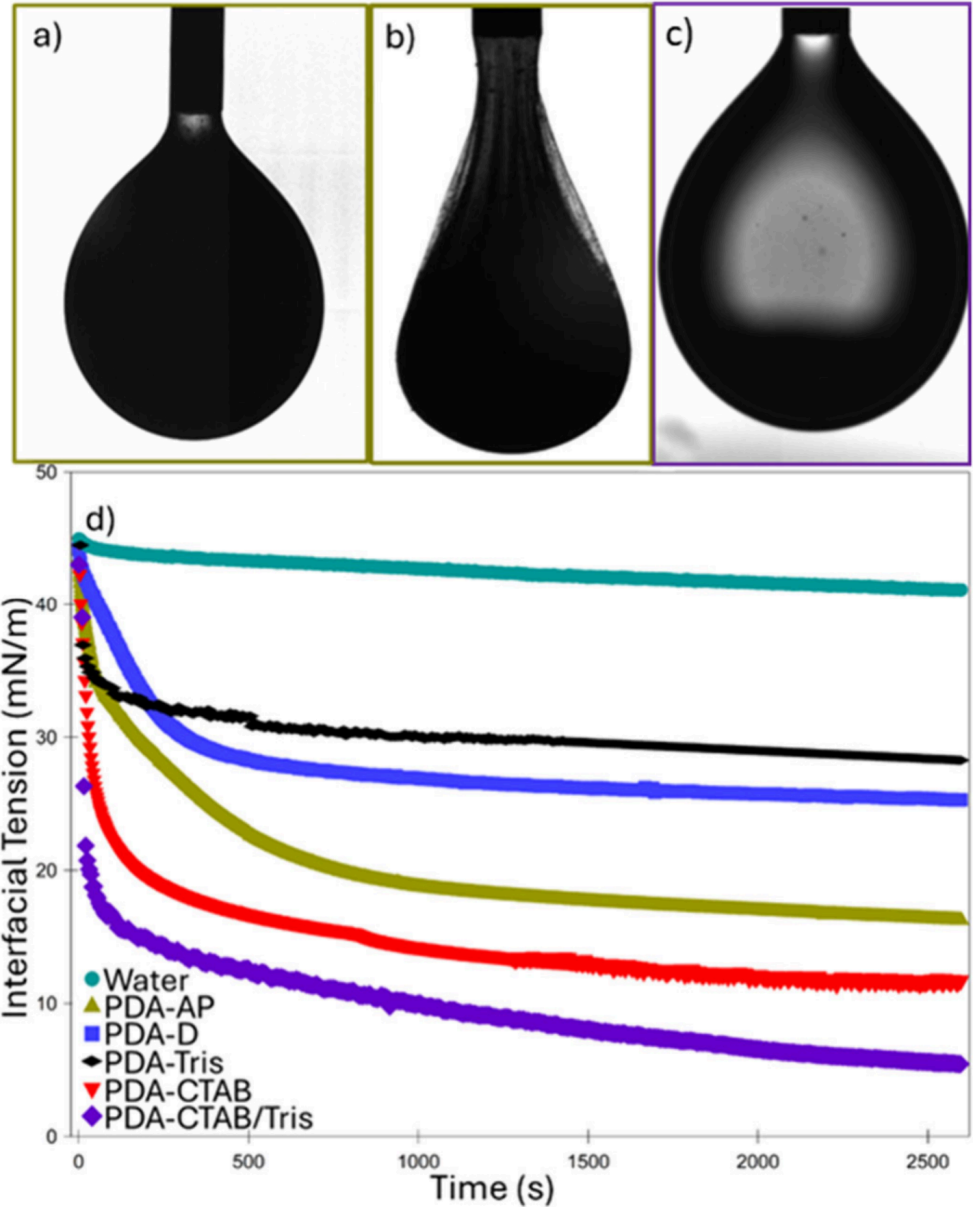
Pendent drop tensiometry of polydopamine particles dispersed in an aqueous phase suspended in a hexadecane oil phase. (a) Photograph of polydopamine particle dispersion as prepared in the emulsion (PDA-AP) at time 0 immediately after suspension in oil and (b) after 2 h demonstrating viscoelastic film formation. It is important to note that no aqueous phase was withdrawn for this film to be visible. (c) Photograph of dialyzed polydopamine particles after addition of CTAB and 0.34 M Tris-HCl (PDA-CTAB/Tris) after 1h (no film apparent). (d) Interfacial tension data of aqueous droplets formed in hexadecane for pure water, PDA-AP suspension, dialyzed polydopamine particle (PDA-D) suspension, dialyzed polydopamine particles dispersed in 0.34 M Tris-HCl (PDA-Tris), dialyzed particles in 0.4 mM CTAB (PDA-CTAB) and PDA-CTAB/Tris. Videos are available in Supporting Information.

The PDA particles formed during the polymerization process appear to form a strong film on the droplet surface, leading to prolonged resistance to coalescence after the polymerization/coalescence process is complete. Thus, interfacial tension measurements were undertaken to investigate this film and the impact of each of the other components of the system. When using the PDA-AP particle suspension only, the droplets formed in hexadecane initially appeared to adopt the traditional pendent shape (Figure 7a). However, after approximately 400 s, a film was visible at the interface (Figure 7). At this point, the Laplacian fit employed by the instrument software was unable to accurately fit the droplet, which no longer possessed a pendent shape.^54^ When left to age for several hours, the film appeared to thicken and began to deform under the weight of the internalized liquid (Figure 7b). This indicated the strong adsorption of particles at the liquid/liquid interface and the formation of an elastic shell. Often in such experiments, this elastic shell is only visible upon droplet compression, but spontaneous wrinkling in this case suggests that additional interactions are reinforcing the interfacial particle network, potentially cross-linking by the aforementioned dissolved PDA polymer or oligomers.^24,55^

This hypothesis is further supported by examination of CryoSEM images in Figure 2c-d where particles are seen to be embedded in a film, rather than organized discretely at the interface. Furthermore, the interfacial tension measurement of the PDA suspension with CTAB and Tris added (Figure 7c) which had been prepared at the same CTAB and Tris-HCl concentration as PDA-AP and the emulsion post dialysis, exhibits different behavior. No film is visible in these experiments, and the interfacial tension (Figure 7d) is observed to drop at a much faster rate, which is characteristic of surfactant adsorption rather than particle adsorption. Indeed, on retraction of the droplet, no buckling or deformation was observed, indicating either the lack of, or weakly adsorbed particles at the O/W interface. Similarly, the PDA-CTAB system demonstrates very similar behavior, but is slightly less effective at reducing the interfacial tension, potentially due to the Tris-HCl screening electrostatic repulsion between the charged CTAB head groups at the interface, resulting in a higher surfactant coverage.^56^ This contrasts with the PDA-Tris sample, which showed the least interfacial activity. Visually, a pendent droplet was formed; however, the interfacial tension measurement exhibited unusual behavior. The measured value appeared to decrease relatively quickly, but the total change in interfacial tension was minimal (only 10 mN/m). These particles were not sufficiently surface-active owing to the increase in pH provided by the Tris-HCl and consequent surface charge. Indeed, this is in agreement with the large zeta potential of these particles measured in Table 2. However, when adding Tris to the initially dialyzed sample, it is possible that some aggregates formed during dialysis and remained in the suspension. As a result, these aggregates would quickly sediment, deforming the droplet, which is misinterpreted by the software as a decrease in IFT.^57^ Finally, the dialyzed PDA particles appeared to be able to lower the interfacial tension in their native state due to the pH of the Milli-Q water being around 6.5, similar to the first average p*K*_a_ of PDA due to the quinone-imine moieties.^58^ However, the catechol groups are still protonated, resulting in a diminished charge and increased amphiphilicity (Table 2), when compared to the other samples, which after Tris-HCl addition are at pH 8.6 (close to p*K*_a_ of the PDA catechol groups–8.9).^58^ At this point, significant deprotonation will take place, resulting in an increased negative charge. Indeed, only dialyzed PDA was able to demonstrate film wrinkling on droplet volume reduction (Video S2) and is reported to be sufficiently surface active to stabilize an emulsion.^41^

**Figure 8:**
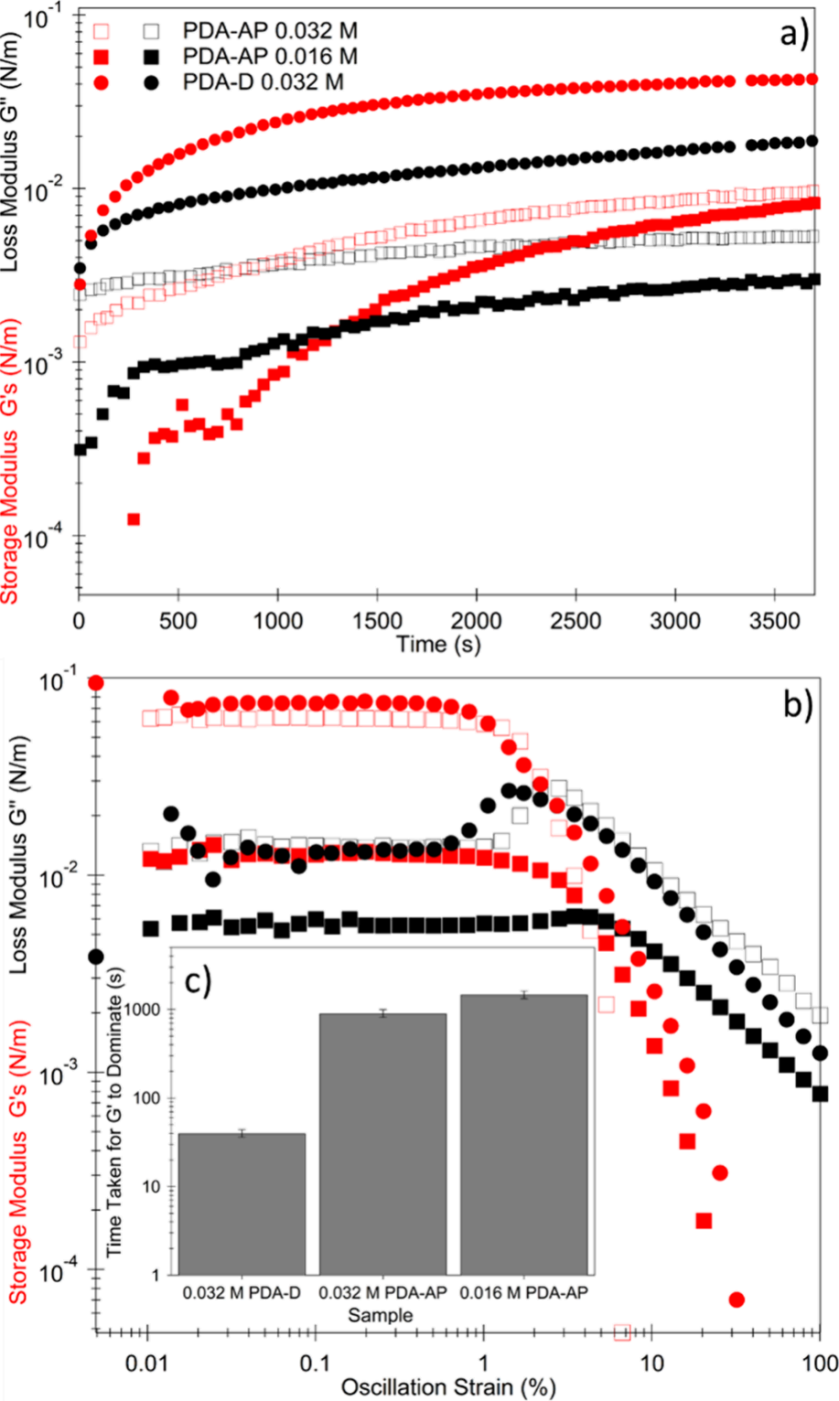
Interfacial properties of the PDA/CTAB–hexadecane film. a) Oscillatory measurements performed on film at 0.5 Hz at a strain amplitude of 1% for PDA-AP at 0.032 and 0.016 M nominal concentration. b) Strain amplitude measurements performed on film after oscillatory measurements (2 h). c) time taken for *G*′ to dominate during oscillatory measurements for each studied sample, error bars are standard error between repeats.

Interfacial Shear Rheology (IFSR) measurements allowed for the monitoring of film formation in situ. The storage (or elastic) modulus *G*′, and loss (or viscous) modulus *G*″ were studied for all the PDA samples along with the interfacial tension measurements; however, only PDA-AP and PDA-D presented a *G*′. This indicated that only samples PDA-AP and PDA-D offered rheological response characteristics of particle films. The response provided by all the other samples was instead characteristic of surfactant stabilized interfaces, which possess only Gibbs elasticity.^59^ These findings were also concordant with the interfacial tension measurements in Figure 7 and the larger positive zeta potential described in Table 2. IFSR was also used to investigate the impact of PDA-AP concentration on film formation behavior (Figure 8). For both concentrations of PDA-AP, *G*″ initially dominates, indicating that the interface is acting as a fluid and flows under the applied strain.^60−62^ However, over time *G*′ increases and reaches a characteristic crossover point with *G*′′ transitioning to an elastic interface, likely due to an increasing number of particles adsorbed at the interface.^63−65^ At this point, the interface no longer flows like a liquid as interparticle, and particle-interface interactions begin to dominate. Beyond the crossover point, *G*′ continues to increase over time, eventually reaching a maximum. A strain sweep was performed on each of the samples after allowing film formation for 2 h. For samples studied at low shear stress (strain <2%), the interfacial moduli were independent of applied strain, indicative of a linear viscoelastic region dominated by the solid-like behavior of the PDA-AP film. However, at a critical strain, *G*′ begins to decrease and crosses with *G*″ as the interface begins to yield and flow like a fluid.^61^ Comparing the behavior of the different concentrations of PDA-AP, it can be seen from Figure 8 that the critical strain required to force the particle-laden interface to yield increases as a function of PDA-AP concentration. Furthermore, the time taken for *G*′ to become dominant increases as the concentration of the particles is decreased. This indicates that the time taken for the elastic film to form is inversely proportional to the concentration of particles present in the aqueous phase, while the yield strain is proportional after initial film formation.

### Polypyrrole/SDS Emulsions

To demonstrate that this coalescence mechanism is not restricted to a single combination of stabilizers, a second system was explored. Pyrrole can be readily polymerized into polypyrrole in a number of ways notably using FeCl_3_ or metal (usually Pd or Pt) salts. The metals oxidize the pyrrole monomer and reduce to FeCl_2_ and Pd/Pt nanoparticles, respectively.^26,66,67^ In this case, we chose to use chloroplatinic acid which allows for formation of polypyrrole through chemical oxidative polymerization. In this system, the polypyrrole polymer forms both at the interface and in the water phase, owing to partitioning of the pyrrole monomer between both phases (LogP = 0.8). In the aqueous phase, the polypyrrole polymer is insoluble and thus precipitates into particles, which can contain platinum nanoparticles within them as a result of the corresponding reduction reaction of choloroplatinic acid. Here we refer to these particles as PPy-Pt.^26,68^

**Figure 9:**
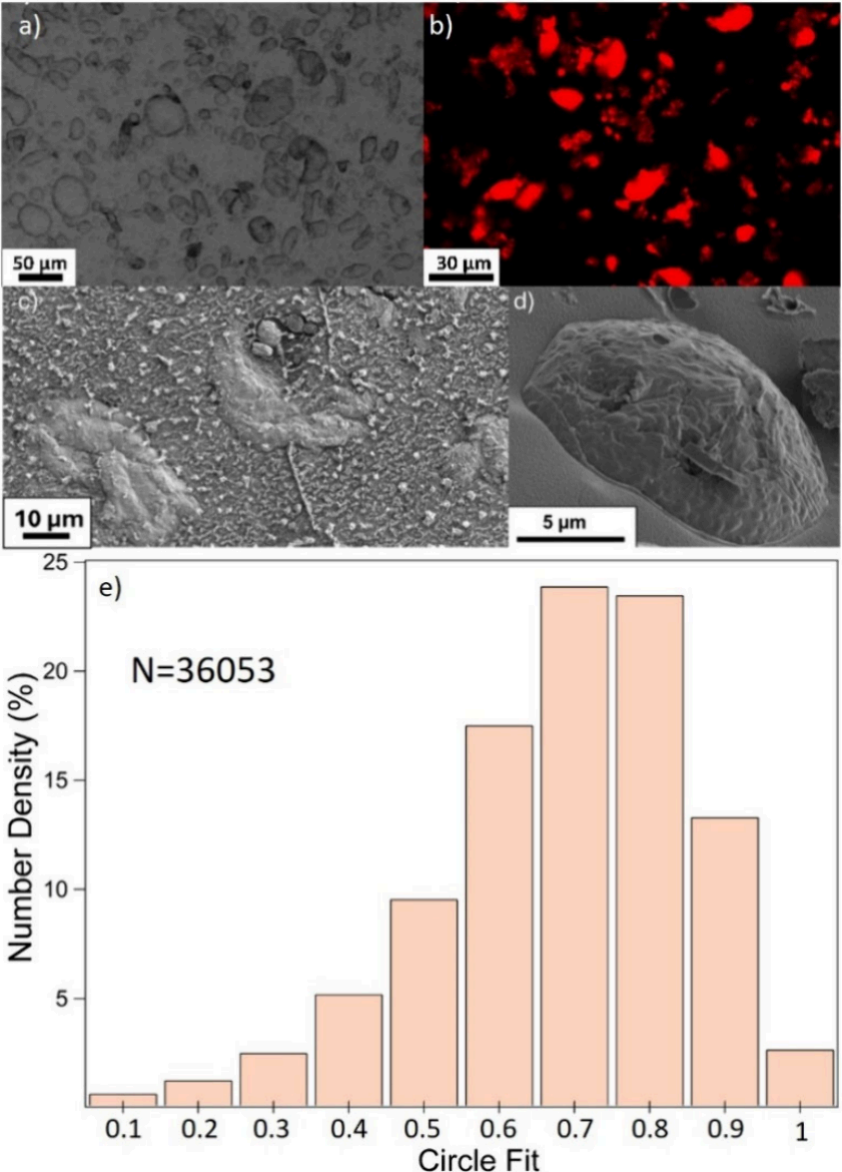
Micrographs and circle fit analysis of nonspherical PPy-Pt/SDS emulsions prepared using 150 mM H_2_PtCl_6_ a) optical micrograph emulsion. b) Repeated emulsion stained with Nile Red under fluorescence using confocal laser scanning microscopy. c,d) CryoSEM micrographs of nonspherical emulsions. e) Circle fit distribution, where N is the number of droplets analyzed.

This process can be compared to the PDA/CTAB system as, in this case, the metal salt both induces an increased coalescence rate of the emulsion initially stabilized by SDS and also initiates the polymerization and PPy-Pt particle formation. Figure 9a-b reveals the presence of a large number of nonspherical droplets in this system resulting from 7 days of coalescence and polymerization. The fundamental mechanism of arrested coalescence is the same as that described for the PDA system, relying on particle formation and interaction with the surfactant. Specifically, the positively charged amine of the PPy may interact with the anionic SDS (PPy-Pt/SDS).^69,70^ The key difference between these two systems is found in the longer polymerization time of pyrrole, as well as the partitioning of the pyrrole monomer in both phases, resulting in direct polymerization at the interface as well as in the bulk. This likely explains the slightly smoother appearance of the PPy-Pt/SDS emulsions seen in Figure 9a-d with fewer discrete particles at the interface. When characterizing the PPy-Pt/SDS emulsion at the end of the process, it was found that the sample also exhibits significant nonsphericity (Figure 9e). An interesting consequence of this formulation process is the potential to further exploit the presence of the Pt nanoparticles, potentially for reaction catalysis or further encapsulation processes using these anisotropic emulsions as a template.^71−73^ The circle fit distribution of the PPy-Pt/SDS emulsion shows a large number of droplets (47%) in the 0.7 and 0.8 bins (nonspherical), and only 20% of the droplets are spherical (≥0.85). However, these systems were not as varied in their interfacial behaviors, which are presented in Figure S11. PPy and PPy-Pt exhibited nearly identical interfacial tension values, with only a small increase in interfacial tension reduction on the addition of SDS. The successful formation of nonspherical droplets using the PPy-Pt/SDS system demonstrated that the anisotropy was not material-specific, but rather due to the mechanism of simultaneous emulsion destabilization and formation of surface-active polymer particles.

## Conclusion

Nonspherical oil/water emulsions were formed using a simple batch process via a process of simultaneous emulsion destabilization and in situ polymeric particle synthesis resulting in interfacial jamming.^16,19^ This mechanism leading to nonspherical emulsion droplets was demonstrated for two separate systems and could be achieved when monomer was present primarily in either the aqueous (dopamine) or oil phase (pyrrole), using either cationic (CTAB) or anionic (SDS) surfactant. The extent of destabilization and degree of nonsphericity was proportional to the concentration of electrolyte added to the emulsion and underpinned by fundamental colloid theories.^31,32^ At electrolyte concentrations of 0.08–0.64 M Tris-HCl in the dopamine system, the number of nonspherical droplets was measured to be 60–80% by number. Shear rate was also found to be a significant factor in the formation of nonspherical emulsions. At an impeller rotation speed of 250 rpm there was a high degree of anisotropy and visibly larger interfacial coverage, while at higher rotation speeds (500 rpm), the number of spherical droplets in the resulting sample increased. This was attributed to higher shear rates allowing for deformation of the droplets and increased collisions, resulting in larger droplets by volume. However, this high shear was also capable of forming daughter droplets, which were inherently spherical, resulting in an increase in the number incidence of sphericity, as confirmed through laser diffraction.^50^ Finally, we investigated the interfacial behavior of the stabilizing particles using pendant drop tensiometry and interfacial shear rheology. Dialyzed polydopamine particles exhibited some interfacial activity, concordant with existing literature;^41^ however, on addition of electrolyte buffer, this activity became diminished. On addition of CTAB the interfacial activity appeared to be dominated by the surfactant, indicated by a fast reduction in interfacial tension. These observations contrasted the observed behavior of particles prepared in a similar manner to those stabilizing the emulsion in the real system, which exhibited a reduced impact of the surfactant, followed by particle adsorption at the interface. However, the measurements of this particular sample may not be reliable due to the formation of a thick viscoelastic film, not adhering to a Laplacian morphology.^54^ This viscoelastic film was studied using interfacial shear rheology with particle concentration being found to impact film formation time and elastic behavior. In contrast to previously reported techniques for producing anisotropic droplets, we required no specialist techniques, methods^14,15,22^ or equipment^4,5,12,13^ to produce micron sized emulsion droplets.^46^ Importantly, this work offers the potential to form nonspherical polymer stabilized droplets using a simple scalable process,^22,23^ which may provide the possibility for increased efficiency in emulsion processing or may act as a template for nonspherical capsules, ideal for increased adhesion in a number of industrial settings such as washing/detergent and agrochemical applications.^9,10^ Indeed, additional work is underway to exploit the surface chemistry of these emulsion droplets, which will allow for subsequent electroless metal deposition and long-term retention and encapsulation of small molecules. The embedded platinum nanoparticles and polydopamine allowing for metal reduction directly on the droplet surface.^72,74^ Once prepared, the relative adhesion of these nonspherical capsules, when compared to spherical capsules could be evaluated using atomic force microscopy and an adhesion cell.^75^ In addition to this encapsulation work, it may be possible to further explore inducing this phenomena in systems where nonadsorbing particles initially dispersed in the emulsion continuous phase could be driven to the interface as a result of addition of electrolyte in the emulsion continuous phase to create conditions where both particle adsorption and droplet coalescence concurrently occur.

## Data Availability

Data will be made available on request.

## Supporting Information Available

Additional experimental details of polypyrrole stabilized emulsions and results of preliminary experiments as well as photographs of reaction vials; ^1^HNMR data along with further analysis of emulsion droplet size and coalescence (PDF)
Video of pendent droplet experiment at 21× speed: Video S1 - PDA AP (MP4)
Video of pendent droplet experiment at 21× speed: Video S2 - PDA-D (MP4)
Video of pendent droplet experiment at 21× speed: Video S3 - PDA-CTAB Tris (MP4)

## References

[ref1] Jerri H. A.; Jacquemond M.; Hansen C.; Ouali L.; Erni P. Suction Caps”: Designing Anisotropic Core/Shell Microcapsules with Controlled Membrane Mechanics and Substrate Affinity. Adv. Funct. Mater. 2016, 26, 6224–6237. 10.1002/adfm.201601563.

[ref2] Dong A.; Wang Y.; Wang D.; Yang W.; Zhang Y.; Ren N.; Gao Z.; Tang Y. Fabrication of hollow zeolite microcapsules with tailored shapes and functionalized interiors. Microporous Mesoporous Mater. 2003, 64, 69–81. 10.1016/S1387-1811(03)00484-0.

[ref3] Bromley K. M.; MacPhee C. E. BslA-stabilized emulsion droplets with designed microstructure. Interface Focus 2017, 7, 20160124 10.1098/rsfs.2016.0124. 28630671 PMC5474033

[ref4] Kudryavtseva V.; Boi S.; Read J.; Guillemet R.; Zhang J.; Udalov A.; Shesterikov E.; Tverdokhlebov S.; Pastorino L.; Gould D. J.; Sukhorukov G. B. Biodegradable Defined Shaped Printed Polymer Microcapsules for Drug Delivery. ACS Appl. Mater. Interfaces 2021, 13, 2371–2381. 10.1021/acsami.0c21607. 33404209

[ref5] Kudryavtseva V.; Bukatin A.; Vyacheslavova E.; Gould D.; Sukhorukov G. B. Printed asymmetric microcapsules: Facile loading and multiple stimuli-responsiveness. Biomaterials Advances 2022, 136, 212762 10.1016/j.bioadv.2022.212762. 35929328

[ref6] Bago Rodriguez A. M.; Binks B. P. Capsules from Pickering emulsion templates. Curr. Opin. Colloid Interface Sci. 2019, 44, 107–129. 10.1016/j.cocis.2019.09.006.

[ref7] Gulumian M.; Andraos C.; Afantitis A.; Puzyn T.; Coville N. J. Importance of Surface Topography in Both Biological Activity and Catalysis of Nanomaterials: Can Catalysis by Design Guide Safe by Design?. International Journal of Molecular Science 2021, 22, 8347 10.3390/ijms22158347. PMC8348784 34361117

[ref8] Cooley M.; Sarode A.; Hoore M.; Fedosov D. A.; Mitragotri S.; Sen Gupta A. Influence of particle size and shape on their margination and wall-adhesion: implications in drug delivery vehicle design across nano-to-micro scale. Nanoscale 2018, 10, 15350–15364. 10.1039/C8NR04042G. 30080212 PMC6247903

[ref9] Graf P.; Finken R.; Seifert U. Adhesion of microcapsules. Langmuir 2006, 22, 7117–7119. 10.1021/la060803l. 16893199

[ref10] Kudryavtseva V.; Sukhorukov G. B. Features of Anisotropic Drug Delivery Systems. Adv. Mater. 2024, 36, e2307675 10.1002/adma.202307675. 38158786

[ref11] Roh S.; Williams A. H.; Bang R. S.; Stoyanov S. D.; Velev O. D. Soft dendritic microparticles with unusual adhesion and structuring properties. Nat. Mater. 2019, 18, 1315–1320. 10.1038/s41563-019-0508-z. 31611673

[ref12] Spicer P. T.; Caggioni M.; Lenis-Abril J.; Bayles A.V. Non-spherical droplet. Proctor and Gamble. U.S. Patent US9597648B2, Oct. 17, 2013.

[ref13] Lian X.; Liao S.; Han W.; Song C.; Wang Y. Stabilizing Liquid in Precise Nonequilibrium Shapes via Fast Interfacial Polymerization. Small 2023, 19, e2301039 10.1002/smll.202301039. 37069770

[ref14] Cholakova D.; Denkov N.; Tcholakova S.; Lesov I.; Smoukov S. K. Control of drop shape transformations in cooled emulsions. Adv. Colloid Interface Sci. 2016, 235, 90–107. 10.1016/j.cis.2016.06.002. 27389390

[ref15] Denkov N.; Tcholakova S.; Lesov I.; Cholakova D.; Smoukov S. K. Self-shaping of oil droplets via the formation of intermediate rotator phases upon cooling. Nature 2015, 528, 392–395. 10.1038/nature16189. 26649824

[ref16] Binks B.P.; Horozov T. S. Colloidal Particles at Liquid Interfaces; Cambridge University Press 2006.

[ref17] Binks B. P.; Lumsdon S. O. Influence of Particle Wettability on the Type and Stability of Surfactant-Free Emulsions. Langmuir 2000, 16, 8622–8631. 10.1021/la000189s.

[ref18] Hao C.; Xie Z.; Atherton T. J.; Spicer P. T. Arrested Coalescence of Viscoelastic Droplets: Ellipsoid Shape Effects and Reorientation. Langmuir 2018, 34, 12379–12386. 10.1021/acs.langmuir.8b02136. 30239202

[ref19] Pawar A. B.; Caggioni M.; Ergun R.; Hartel R. W.; Spicer P. T. Arrested coalescence in Pickering emulsions. Soft Matter 2011, 7, 7710–7716. 10.1039/c1sm05457k.

[ref20] Golemanov K.; Tcholakova S.; Kralchevsky P. A.; Ananthapadmanabhan K. P.; Lips A. Latex-Particle-Stabilized Emulsions of Anti-Bancroft Type. Langmuir 2006, 22, 4968–4977. 10.1021/la0603875. 16700582

[ref21] Whitby C. P.; Wanless E. J. Controlling Pickering Emulsion Destabilisation: A Route to Fabricating New Materials by Phase Inversion. Materials 2016, 9, 626 10.3390/ma9080626. 28773747 PMC5509044

[ref22] Bon S. A. F.; Mookhoek S. D.; Colver P. J.; Fischer H. R.; van der Zwaag S. Route to stable non-spherical emulsion droplets. Eur. Polym. J. 2007, 43, 4839–4842. 10.1016/j.eurpolymj.2007.09.001.

[ref23] Bala Subramaniam A.; Abkarian M.; Mahadevan L.; Stone H. A. Non-spherical bubbles. Nature 2005, 438, 930 10.1038/438930a. 16355208

[ref24] Cui M.; Emrick T.; Russell T. P. Stabilizing liquid drops in nonequilibrium shapes by the interfacial jamming of nanoparticles. Science 2013, 342, 460–463. 10.1126/science.1242852. 24159042

[ref25] Forth J.; Liu X.; Hasnain J.; Toor A.; Miszta K.; Shi S.; Geissler P. L.; Emrick T.; Helms B. A.; Russell T. P. Reconfigurable Printed Liquids. Adv. Mater. 2018, 30, e1707603 10.1002/adma.201707603. 29573293

[ref26] Takeoka H.; Hamasaki H.; Harada Y.; Nakamura Y.; Fujii S. Synthesis and characterization of polypyrrole-platinum nanocomposite-coated latex particles. Colloid Polym. Sci. 2015, 293, 1483–1493. 10.1007/s00396-015-3534-7.

[ref27] Hoorfar M.; W. Neumann A. Recent progress in axisymmetric drop shape analysis (ADSA). Adv. Colloid Interface Sci. 2006, 121, 25–49. 10.1016/j.cis.2006.06.001. 16854362

[ref28] Saad S. M. I.; Policova Z.; Neumann A. W. Design and accuracy of pendant drop methods for surface tension measurement. Colloids Surf., A 2011, 384, 442–452. 10.1016/j.colsurfa.2011.05.002.

[ref29] Berry J. D.; Neeson M. J.; Dagastine R. R.; Chan D. Y.; Tabor R. F. Measurement of surface and interfacial tension using pendant drop tensiometry. J. Colloid Interface Sci. 2015, 454, 226–237. 10.1016/j.jcis.2015.05.012. 26037272

[ref30] Pawar A. B.; Caggioni M.; Hartel R. W.; Spicer P. T. Arrested coalescence of viscoelastic droplets with internal microstructure. Faraday Discuss. 2012, 158, 341–350. 10.1039/c2fd20029e. 23234175

[ref31] Derjaguin B. V.; Landau L. Theory of the stability of stronglycharged lyophobic sols and of the adhesion of strongly charged particles in solution of electrolytes. Prog. Surf. Sci. 1993, 43, 30–59. 10.1016/0079-6816(93)90013-L.

[ref32] Verwey E.J.W.; Overbeek J.T.G. Theory of the Stability of Lyophobic Colloids: The Interaction of Sol Particles Having an Electric Double Layer; Elsevier Publishing, 1948.

[ref33] Ponzio F.; Ball V. Polydopamine deposition at fluid interfaces. Polym. Int. 2016, 65, 1251–1257. 10.1002/pi.5124.

[ref34] Liu Y.; Ai K.; Lu L. Polydopamine and its derivative materials: synthesis and promising applications in energy, environmental, and biomedical fields. Chem. Rev. 2014, 114, 5057–5115. 10.1021/cr400407a. 24517847

[ref35] Liu P.; Qi C.; Gao Y. CTAB-assisted fabrication of well-shaped PDA-based colloidosomes. Colloid Polym. Sci. 2019, 297, 1301–1311. 10.1007/s00396-019-04547-w.

[ref36] Hunter T. N.; Pugh R. J.; Franks G. V.; Jameson G. J. The role of particles in stabilising foams and emulsions. Adv. Colloid Interface Sci. 2008, 137, 57–81. 10.1016/j.cis.2007.07.007. 17904510

[ref37] Liu Y.; Tourbin M.; Lachaize S.; Guiraud P. Silica nanoparticles separation from water: aggregation by cetyltrimethylammonium bromide (CTAB). Chemosphere 2013, 92, 681–687. 10.1016/j.chemosphere.2013.03.048. 23618346

[ref38] Ma X.-K.; Lee N.-H.; Oh H.-J.; Kim J.-W.; Rhee C.-K.; Park K.-S.; Kim S.-J. Surface modification and characterization of highly dispersed silica nanoparticles by a cationic surfactant. Colloids Surf., A 2010, 358, 172–176. 10.1016/j.colsurfa.2010.01.051.

[ref39] Kora A. J.; Manjusha R.; Arunachalam J. Superior bactericidal activity of SDS capped silver nanoparticles: Synthesis and characterization. Materials Science and Engineering: C 2009, 29, 2104–2109. 10.1016/j.msec.2009.04.010.

[ref40] Lai Y.-C.; Lai C.-S.; Tai J.-T.; Nguyen T. P.; Wang H.-L.; Lin C.-Y.; Tsai T.-Y.; Ho H.-C.; Wang P.-H.; Liao Y.-C.; Tsai D.-H. Understanding ligand–nanoparticle interactions for silica, ceria, and titania nanopowders. Advanced Powder Technology 2015, 26, 1676–1686. 10.1016/j.apt.2015.10.005.

[ref41] Nishizawa N.; Kawamura A.; Kohri M.; Nakamura Y.; Fujii S. Polydopamine Particle as a Particulate Emulsifier. Polymers 2016, 8, 62 10.3390/polym8030062. 30979157 PMC6432528

[ref42] Clegg P. S.; Herzig E. M.; Schofield A. B.; Horozov T. S.; Binks B. P.; Cates M. E.; Poon W. C. K. Colloid-stabilized emulsions: behaviour as the interfacial tension is reduced. J. Phys.: Condens. Matter 2005, 17, S3433–S3438. 10.1088/0953-8984/17/45/031.

[ref44] Knox S. T.; Parkinson S.; Stone R.; Warren N. J. Benchtop flow-NMR for rapid online monitoring of RAFT and free radical polymerisation in batch and continuous reactors. Polym. Chem. 2019, 10, 4774–4778. 10.1039/C9PY00982E.

[ref45] Ball V.; Frari D. D.; Toniazzo V.; Ruch D. Kinetics of polydopamine film deposition as a function of pH and dopamine concentration: insights in the polydopamine deposition mechanism. J. Colloid Interface Sci. 2012, 386, 366–372. 10.1016/j.jcis.2012.07.030. 22874639

[ref46] Tyowua A. T.; Targema M.; Ubuo E. E. Salt-induced edible anisotropic Pickering emulsion droplets. J. Dispersion Sci. Technol. 2023, 44, 1979–1990. 10.1080/01932691.2022.2055564.

[ref47] Gould J.; Garcia-Garcia G.; Wolf B. Pickering Particles Prepared from Food Waste. Materials 2016, 9, 791 10.3390/ma9090791. 28773909 PMC5457084

[ref48] Tolstoguzov V. B.; Mzhel’sky A. I.; Gulov V. Y. Deformation of emulsion droplets in flow. Colloid Polym. Sci. 1974, 252, 124–132. 10.1007/BF01555536.

[ref49] Karam H. J.; Bellinger J. C. Deformation and Breakup of Liquid Droplets in a Simple Shear Field. Industrial & Engineering Chemistry Fundamentals 1968, 7, 576–581. 10.1021/i160028a009.

[ref50] Whitby C. P.; Fischer F. E.; Fornasiero D.; Ralston J. Shear-induced coalescence of oil-in-water Pickering emulsions. J. Colloid Interface Sci. 2011, 361, 170–177. 10.1016/j.jcis.2011.05.046. 21658702

[ref51] Atkin R.; Craig V. S.; Wanless E. J.; Biggs S. Mechanism of cationic surfactant adsorption at the solid-aqueous interface. Adv. Colloid Interface Sci. 2003, 103, 219–304. 10.1016/S0001-8686(03)00002-2. 12781966

[ref52] Ponzio F.; Bertani P.; Ball V. Role of surfactants in the control of dopamine-eumelanin particle size and in the inhibition of film deposition at solid-liquid interfaces. J. Colloid Interface Sci. 2014, 431, 176–179. 10.1016/j.jcis.2014.06.025. 24997433

[ref53] Cihanoglu A.; Schiffman J. D.; Alsoy Altinkaya S. Biofouling-Resistant Ultrafiltration Membranes via Codeposition of Dopamine and Cetyltrimethylammonium Bromide with Retained Size Selectivity and Water Flux. ACS Appl. Mater. Interfaces 2022, 14, 38116–38131. 10.1021/acsami.2c05844. 35947443 PMC9412966

[ref54] Ferri J. K.; Fernandes P. A. L.; McRuiz J. T.; Gambinossi F. Elastic nanomembrane metrology at fluid–fluid interfaces using axisymmetric drop shape analysis with anisotropic surface tensions: deviations from Young–Laplace equation. Soft Matter 2012, 8, 10352–10359. 10.1039/c2sm26604k.

[ref55] Hemmatpour H.; De Luca O.; Crestani D.; Stuart M. C. A.; Lasorsa A.; van der Wel P. C. A.; Loos K.; Giousis T.; Haddadi-Asl V.; Rudolf P. New insights in polydopamine formation via surface adsorption. Nat. Commun. 2023, 14, 664 10.1038/s41467-023-36303-8. 36750751 PMC9905603

[ref56] Qazi M. J.; Schlegel S. J.; Backus E. H. G.; Bonn M.; Bonn D.; Shahidzadeh N. Dynamic Surface Tension of Surfactants in the Presence of High Salt Concentrations. Langmuir 2020, 36, 7956–7964. 10.1021/acs.langmuir.0c01211. 32545966 PMC7366510

[ref57] Delahaije R.; Sagis L. M. C.; Yang J. Impact of Particle Sedimentation in Pendant Drop Tensiometry. Langmuir 2022, 38, 10183–10191. 10.1021/acs.langmuir.2c01193. 35943288 PMC9404539

[ref58] Chen F.; Xing Y.; Wang Z.; Zheng X.; Zhang J.; Cai K. Nanoscale Polydopamine (PDA) Meets pi-pi Interactions: An Interface-Directed Coassembly Approach for Mesoporous Nanoparticles. Langmuir 2016, 32, 12119–12128. 10.1021/acs.langmuir.6b03294. 27933877

[ref59] Lucassen-Reynders E. H.; Cagna A.; Lucassen J. Gibbs elasticity, surface dilational modulus and diffusional relaxation in nonionic surfactant monolayers. Colloids Surf., A 2001, 186, 63–72. 10.1016/S0927-7757(01)00483-6.

[ref60] Varanasi S.; Henzel L.; Mendoza L.; Prathapan R.; Batchelor W.; Tabor R.; Garnier G. Pickering Emulsions Electrostatically Stabilized by Cellulose Nanocrystals. Frontiers in Chemistry 2018, 6, 409 10.3389/fchem.2018.00409. 30283771 PMC6157443

[ref61] Krägel J.; Derkatch S. R. Interfacial shear rheology. Curr. Opin. Colloid Interface Sci. 2010, 15, 246–255. 10.1016/j.cocis.2010.02.001. 18823871

[ref62] Mendoza A. J.; Guzman E.; Martinez-Pedrero F.; Ritacco H.; Rubio R. G.; Ortega F.; Starov V. M.; Miller R. Particle laden fluid interfaces: dynamics and interfacial rheology. Adv. Colloid Interface Sci. 2014, 206, 303–319. 10.1016/j.cis.2013.10.010. 24200090

[ref63] Yu K.; Zhang H.; Biggs S.; Xu Z.; Cayre O. J.; Harbottle D. The rheology of polyvinylpyrrolidone-coated silica nanoparticles positioned at an air-aqueous interface. J. Colloid Interface Sci. 2018, 527, 346–355. 10.1016/j.jcis.2018.05.035. 29804004

[ref64] Fuller G. G.; Vermant J. Complex fluid-fluid interfaces: rheology and structure. Annu. Rev. Chem. Biomol. Eng. 2012, 3, 519–543. 10.1146/annurev-chembioeng-061010-114202. 22541047

[ref65] Krishnaswamy R.; Majumdar S.; Ganapathy R.; Agarwal V. V.; Sood A. K.; Rao C. N. Interfacial rheology of an ultrathin nanocrystalline film formed at the liquid/liquid interface. Langmuir 2007, 23, 3084–3087. 10.1021/la063236a. 17300211

[ref66] DeArmitt C.; Armes S. P. Colloidal dispersions of surfactant-stabilized polypyrrole particles. Langmuir 1993, 9, 652–654. 10.1021/la00027a007.

[ref67] Hazarika J.; Kumar A. Controllable synthesis and characterization of polypyrrole nanoparticles in sodium dodecylsulphate (SDS) micellar solutions. Synth. Met. 2013, 175, 155–162. 10.1016/j.synthmet.2013.05.020.

[ref68] Fujii S.; Matsuzawa S.; Nakamura Y.; Ohtaka A.; Teratani T.; Akamatsu K.; Tsuruoka T.; Nawafune H. Synthesis and characterization of polypyrrole-palladium nanocomposite-coated latex particles and their use as a catalyst for Suzuki coupling reaction in aqueous media. Langmuir 2010, 26, 6230–6239. 10.1021/la9039545. 20146495

[ref69] Šišáková M.; Asaumi Y.; Uda M.; Seike M.; Oyama K.; Higashimoto S.; Hirai T.; Nakamura Y.; Fujii S. Dodecyl sulfate-doped polypyrrole derivative grains as a light-responsive liquid marble stabilizer. Polym. J. 2020, 52, 589–599. 10.1038/s41428-020-0307-z.

[ref70] Xing S.; Zhao G. Morphology, structure, and conductivity of polypyrrole prepared in the presence of mixed surfactants in aqueous solutions. J. Appl. Polym. Sci. 2007, 104, 1987–1996. 10.1002/app.25912.

[ref71] Horiuchi S.; Nakao Y. Platinum colloid catalyzed etchingless gold electroless plating with strong adhesion to polymers. Surf. Coat. Technol. 2010, 204, 3811–3817. 10.1016/j.surfcoat.2010.04.064.

[ref72] Hitchcock J.; White A. L.; Hondow N.; Hughes T. A.; Dupont H.; Biggs S.; Cayre O. J. Metal-shell nanocapsules for the delivery of cancer drugs. J. Colloid Interface Sci. 2020, 567, 171–180. 10.1016/j.jcis.2019.12.018. 32045739

[ref73] Lobel B. T.; Baiocco D.; Al-Sharabi M.; Routh A. F.; Zhang Z.; Cayre O. J. Current Challenges in Microcapsule Designs and Microencapsulation Processes: A Review. ACS Appl. Mater. Interfaces 2024, 16, 40326–40355. 10.1021/acsami.4c02462. 39042830 PMC11311140

[ref74] Nocera G. M.; Ben M’Barek K.; Bazzoli D. G.; Fraux G.; Bontems-Van Heijenoort M.; Chokki J.; Georgeault S.; Chen Y.; Fattaccioli J. Fluorescent microparticles fabricated through chemical coating of O/W emulsion droplets with a thin metallic film, RSC. Advances 2014, 4, 11564–11568. 10.1039/c3ra47063f.

[ref75] He Y.; Bowen J.; Andrews J. W.; Liu M.; Smets J.; Zhang Z. Adhesion of perfume-filled microcapsules to model fabric surfaces. J. Microencapsulation 2014, 31, 430–439. 10.3109/02652048.2013.871359. 24697187

