# Supplementary material for: Nonspherical Particle Stabilized Emulsions Formed through Destabilization and Arrested Coalescence
Source: Langmuir. 2024 Dec 26;41(1):550–62. doi: 10.1021/acs.langmuir.4c03812 (PMC11736840; doi:10.1021/acs.langmuir.4c03812)
Supplement: Supplementary file 1 — la4c03812_si_001.pdf [file la4c03812_si_001.pdf]

Supporting Information

Non-spherical particle stabilised emulsions formed through destabilisation and arrested coalescence

Benjamin T. Lobel[1a]*, Daniele Baiocco[2], Mohammed Al-Sharabi[3], Alexander F. Routh[3], Zhibing Zhang[2], Olivier J. Cayre[1]*

[1]School of Chemical and Process Engineering, University of Leeds, Leeds, LS2 9JT, United Kingdom

[2]School of Chemical Engineering, University of Birmingham, Birmingham, B15 2TT, United Kingdom

[3]Department of Chemical Engineering and Biotechnology, University of Cambridge, Cambridge, CB3 0AS, United Kingdom

[a]Current Address: School of Mathematics, Statistics, Chemistry and Physics, Murdoch University, Murdoch 6150, Australia

*Corresponding Author: b.t.lobel@murdoch.edu.au

*Corresponding Author: o.j.cayre@leeds.ac.uk

Preliminary Experiments Varying Monomer Quantity & Emulsion Vials

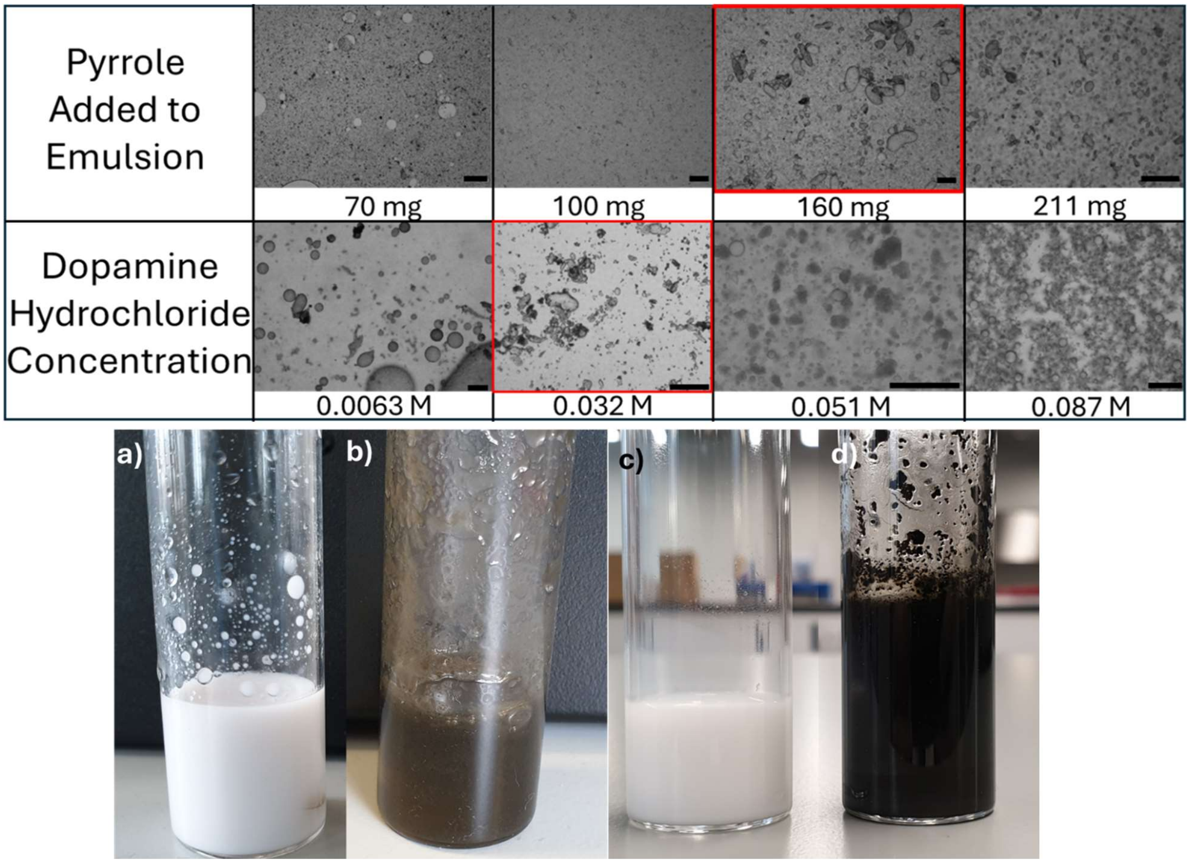

Figure S1. Preliminary emulsion experiments using varied monomer amounts. Top: Micrographs of PPy-Pt/SDS emulsions formed with increasing masses of pyrrole added to SDS stabilised emulsion and micrographs of PDA/CTAB emulsions using increasing concentrations of dopamine hydrochloride. All scale bars are 100 µm. Red boxes indicate amounts used in emulsions discussed in main manuscript. All emulsions were prepared as outlined in the main manuscript except for the varied monomer amounts. Bottom: Digital photographs of emulsions. a) CTAB stabilised hexadecane oil in water emulsion with dopamine hydrochloride in continuous phase. b) hexadecane oil in water emulsion after addition of Tris-HCl buffer and 24 h polymerisation of dopamine to polydopamine resulting in particle-stabilised emulsion. c) SDS stabilised hexadecane oil in water emulsion. d) hexadecane oil in water emulsion after addition of pyrrole, chloroplatinic acid and 7-day polymerisation of pyrrole to polypyrrole resulting in particle stabilised emulsion.

Polypyrrole-Platinum/SDS Non-Spherical Emulsion Formation

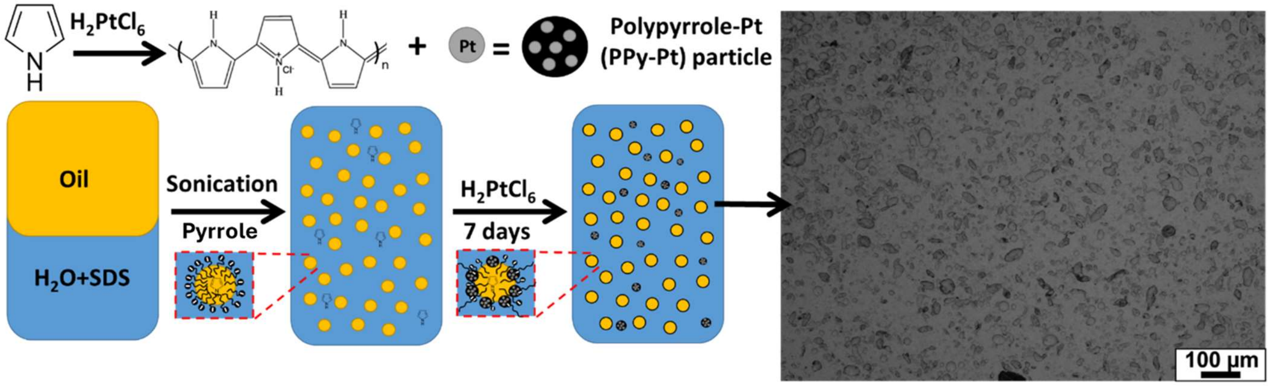

Figure S2. Schematic of anisotropic emulsion formation. Synthetic method based on work by Takeoka et al.[1] Initial SDS stabilised oil/water emulsion formed via sonication. Pyrrole is added after emulsification and allowed to stir for 1 h to allow for partitioning into the oil phase. After 1 h chloroplatinic acid solution is added to initiated chemical oxidative polymerisation and simultaneous electrostatically driven destabilisation (coalescence). Polypyrrole particles are then formed in the continuous phase, and at the interface imbedded with Pt particles as a redox product. This results in non-spherical emulsion droplets (right). If the ratio of polymer, surfactant or oil is changed then this process does not proceed, resulting in spherical droplets.

Sizing of Polydopamine Nanoparticles

After preparation as outlined in §2.5 of the manuscript PDA-AP particles were diluted 1:100 and added to a 1 cm cuvette. The particle size distribution was then determined using a dynamic light scattering (DLS, Malvern Zetasizer Ultra) using backscatted. Particles were determined to have a Z-average diameter of 770 nm and an intensity based mean diameter of 604 nm.

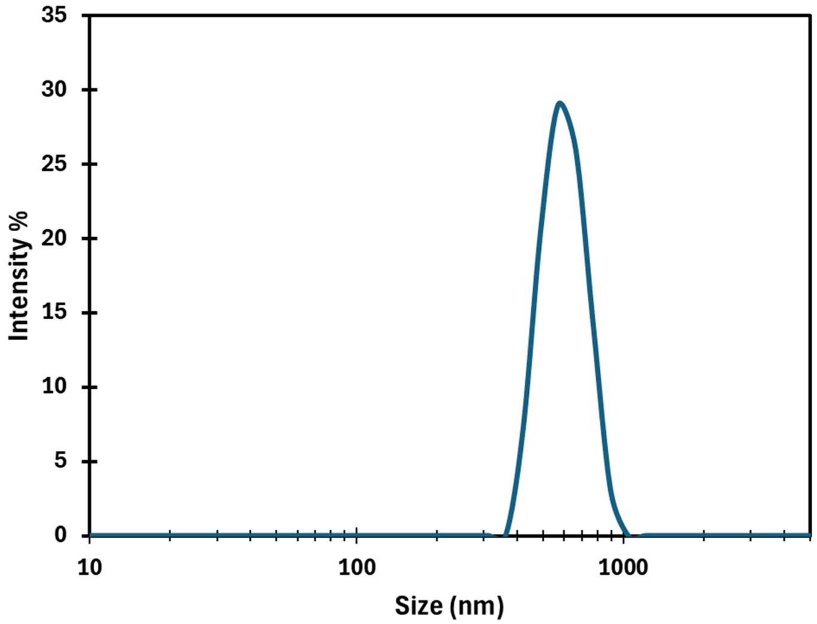

Figure S3. Size distribution of PDA-AP nanoparticles as determined by dynamic light scattering.

FlowCam Analysis

Emulsions were imaged in reverse flow configuration as outlined in the main manuscript. Once imaging was completed, individual droplet images were collected. These images were then filtered using the "Edge Gradient" function setting the lower threshold to 150 and upper to a maximum of 255. The remaining droplets were then analysed using the "Circle fit" function to ascertain their non-sphericity.

From the instrument manual:

Edge Gradient: Average intensity of the pixels making up the outside border of a particle after a deconvolution filter has been applied to the raw image.

Meaning that if the pixel intensity at a particle/droplet edge was weak or blurred the image was removed from the set, this was performed to limit issues caused by depth of field of the recording camera that would result in blurred images and inaccurate measurements.

Circle Fit: Deviation of the particle edge from a best-fit circle normalized to the range [0,1] where a perfect fit has a value of 1. 1 is the value for a perfect circle, values near zero are for particles that are not at all circular

Meaning a circle is projected on to the image of the particle or droplet captured, and the perimeter of the droplet/particle is measured. The deviation from the perimeter line of the particle/droplet from the circle of best fit is then calculated, with 0.1 being the most deviation and 1 being completely congruent with the projected circle.

It is important to note that due to the edge gradient function the perimeter line of any droplet would be easily discernible by the software and the circle fit function could function as intended. Our droplets were categorised as being spherical if they presented with a circle fit greater than 0.85. All droplets were then placed in bins of 0.1 as presented in the main manuscript.

^{1}H NMR Analysis

NMR was used to track the polymerisation of the dopamine to polydopamine. This was accomplished by preparing a solution of 3 mL 0.05M dopamine hydrochloride, combined with 100 µL of 18 mM CTAB. NMR of this solution was obtained using a 60 Hz benchtop NMR spectrometer (Magritek) and denotes time 0 as shown in Figure S5.[2] After this, 1.6 mL of 1 M Tris-HCl buffer was added to the solution to initiate polymerisation. At specific time intervals 1 mL of sample was taken and passed through a 0.22 µm filter to remove any formed PDA particles before being reanalysed (Figure S3). A calibration curve was then constructed of dopamine hydrochloride samples at increasing concentrations, using the peak present at 6.85 ppm to quantify the monomer remaining in solution (Figures S9). This subsequently allowed for the determination of remaining monomer present during polymerisation and thus reaction kinetics (Figure S6).

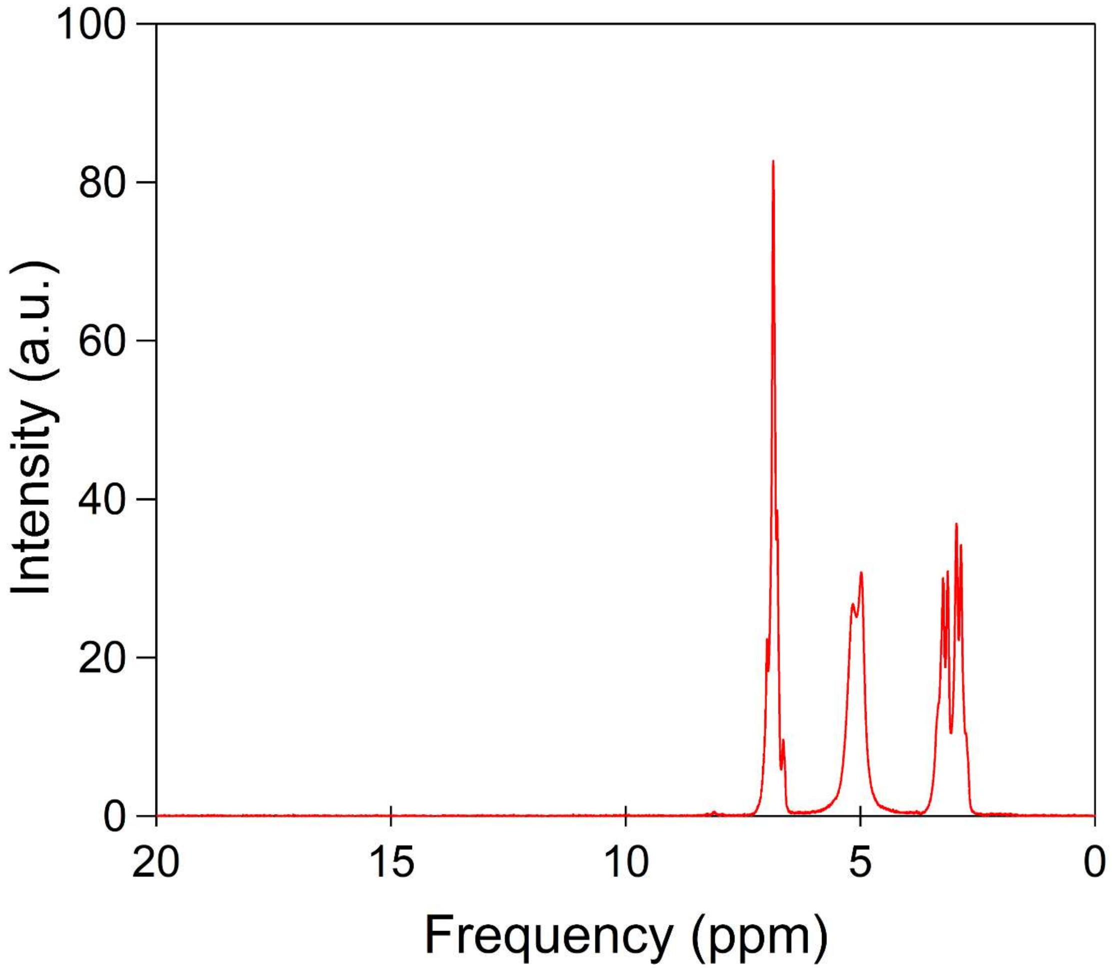

Figure S4. ^{1}HNMR spectrum of 1 M dopamine hydrochloride solution.

^{1}H NMR of Polymerization of Dopamine to Polydopamine

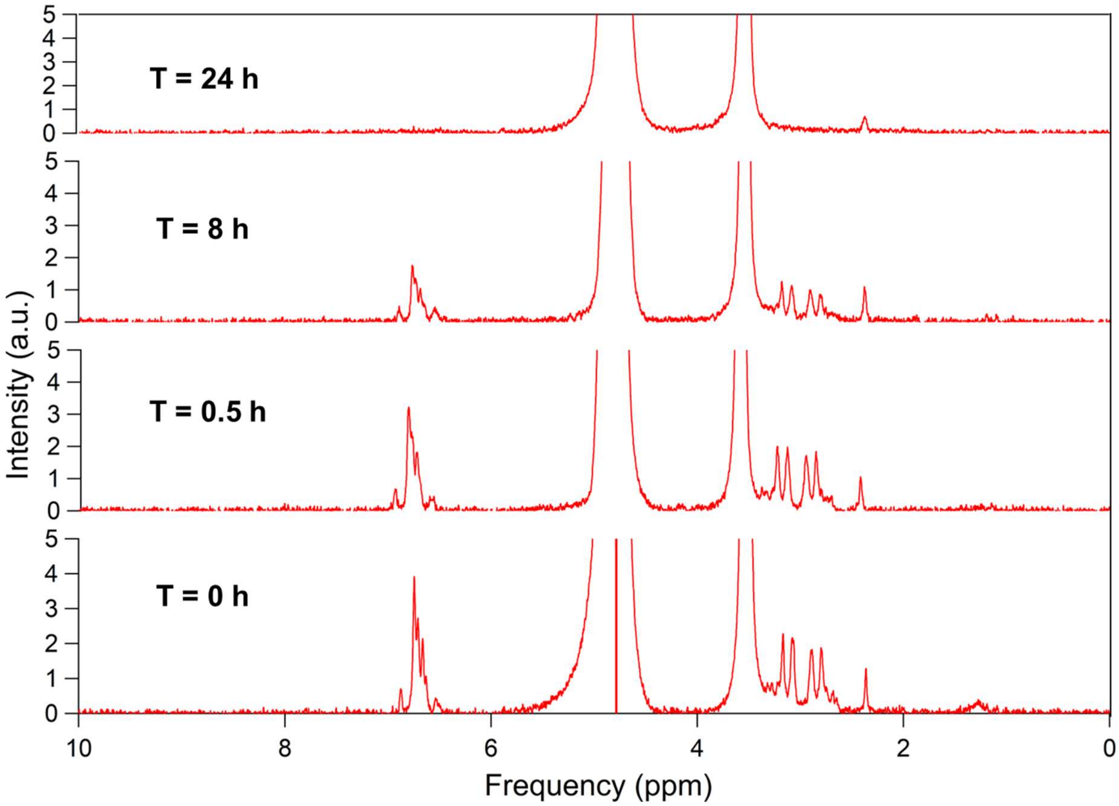

Figure S5. ^{1}HNMR spectra at time intervals during polymerisation. The reduction of the peak at 6.85 ppm can clearly be observed as the polymerisation progresses.

Calibration Curve Used To Determine Remaining Dopamine in Polymerization Solution

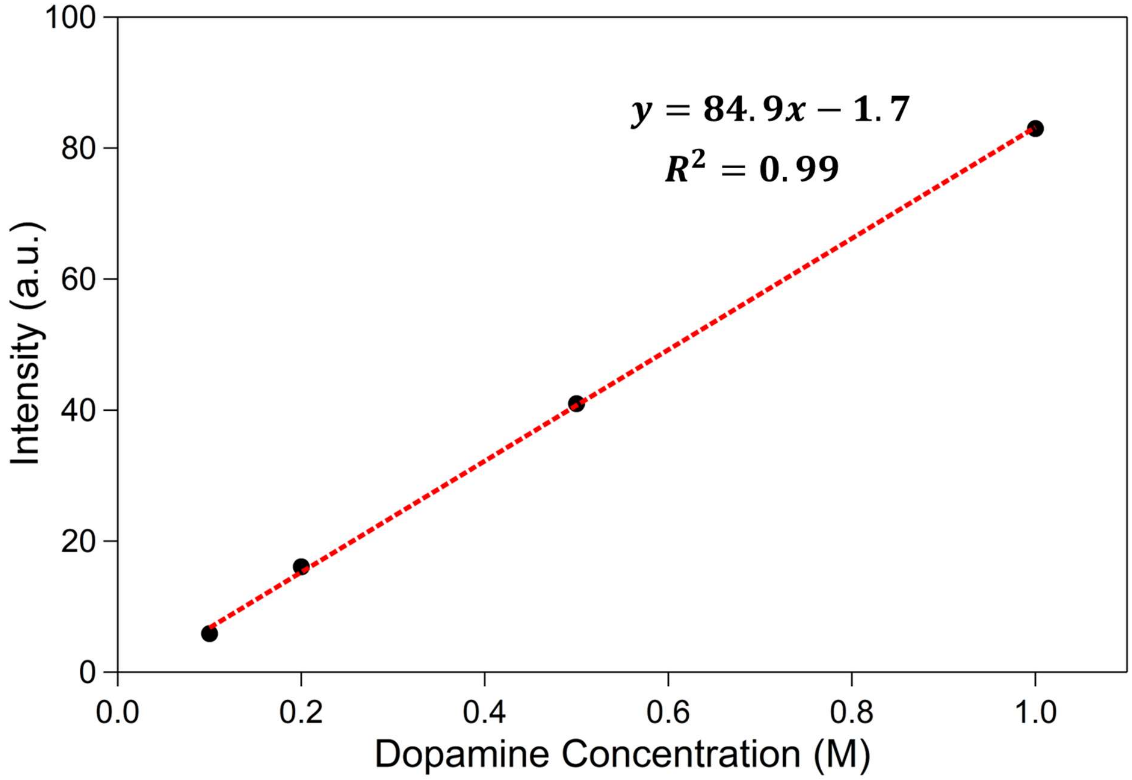

Figure S6. Calibration curve formed from ^{1}HNMR spectra of dopamine hydrochloride samples at increasing concentrations using peak at 6.85 ppm

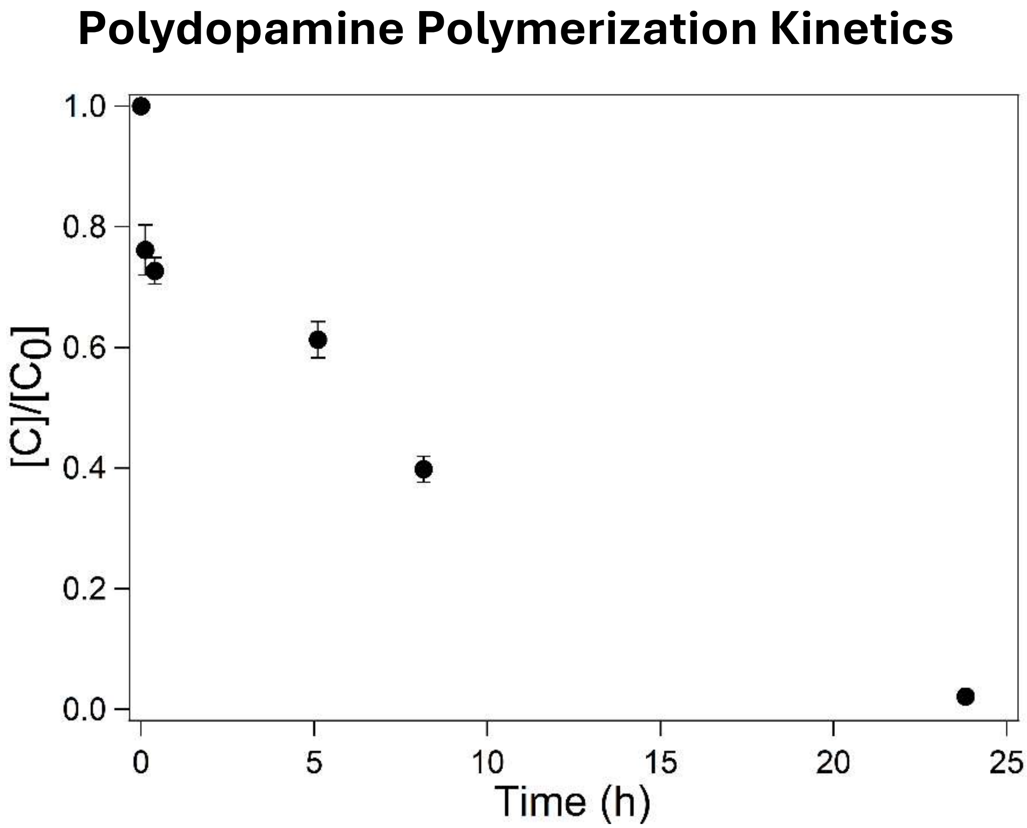

Figure S7. Polymerization kinetics of dopamine hydrochloride to polydopamine as determined by Figures S4-S6. $[C_0]$ denotes the concentration at time = 0 and [C] the concentration of time at sampling as determined from the calibration curve.

Coalescence Kinetics

CTAB stabilised emulsions were formed as outlined in the main manuscript in §2.1 without dopamine hydrochloride. Figure S8 (top) was not treated with Tris-HCl and shows little coalescence over time (3 days). Conversely, Figure S8 (bottom) was treated with 1.6 mL 1 M Tris-HCl, and began to coalesce over time. Measurements were taken at the stated interval using Malvern Mastersizer 3000. Emulsions were added to the hydrodispersion unit until obscuration was sufficient for valid measurement.

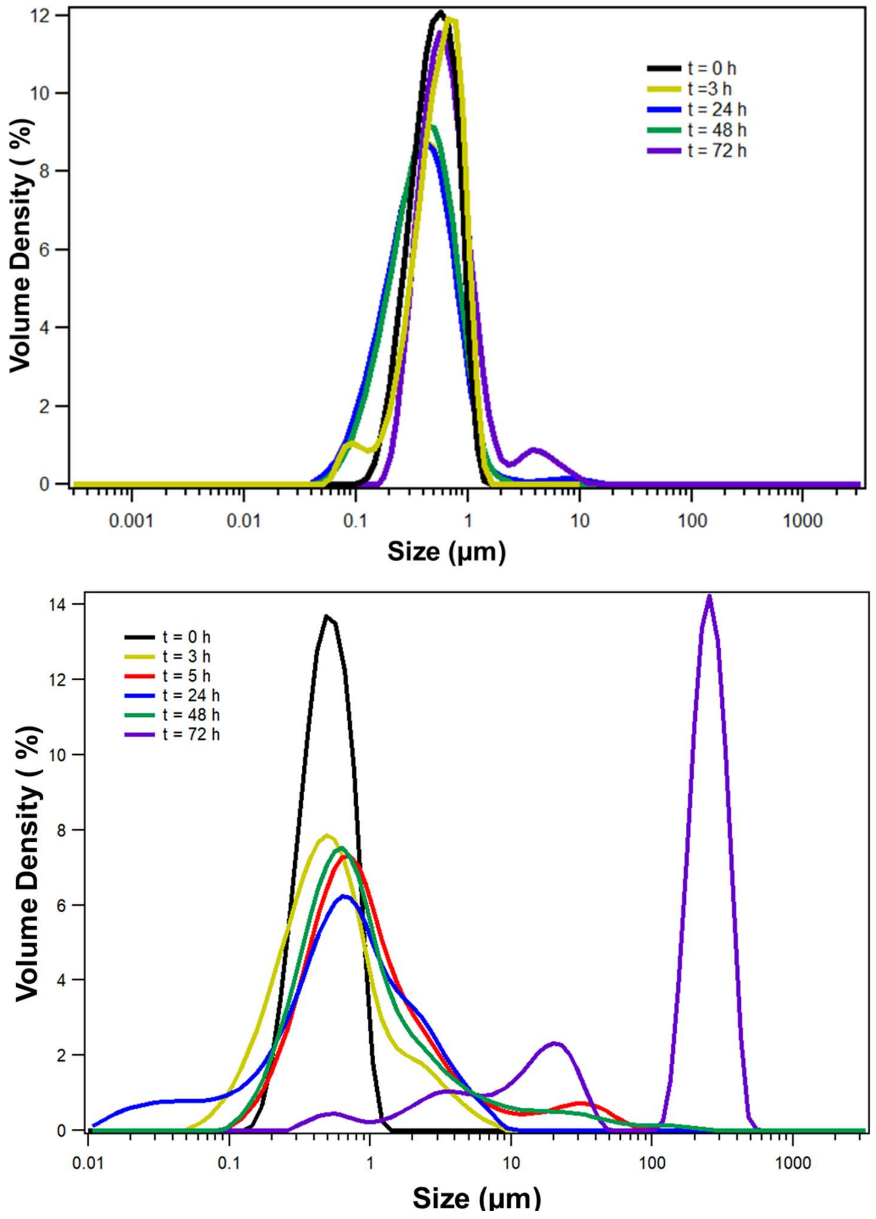

Figure S8. Size distributions of CTAB stabilised emulsions over 72 h. Top: Without the addition of any Tris-HCl Buffer. Bottom: After Addition of 1.6 mL of 1 M Tris-HCl buffer at time 0. Emulsions were prepared as outlined in the main manuscript and measured using laser diffraction (Malvern Mastersizer 3000, equipped with a hydrodispersion unit).

Energy Dispersive X-ray Spectroscopy

During CryoSEM imaging of PPy-Pt/SDS emulsions as outlined in the main manuscript, EDX was used to ensure emulsion droplets were being imaged. The carbon signal was used to identify droplets and was able to visualise the anisotropy of the formed emulsion. It should be noted that the emulsions were centrifuged before imaging and as such loose pyrrole particles should have settled out and not been accessible for CryoSEM sampling. Furthermore, while carbon is also present in the surfactant used (SDS) it would not be expected that individual surfactant molecules or micelles would be able to be observed at this magnification or using this technique. Indeed, even if the surfactant is responsible for the carbon signal – they would primarily be located at the oil/water interface in this case, that is on the surface of an oil droplet.

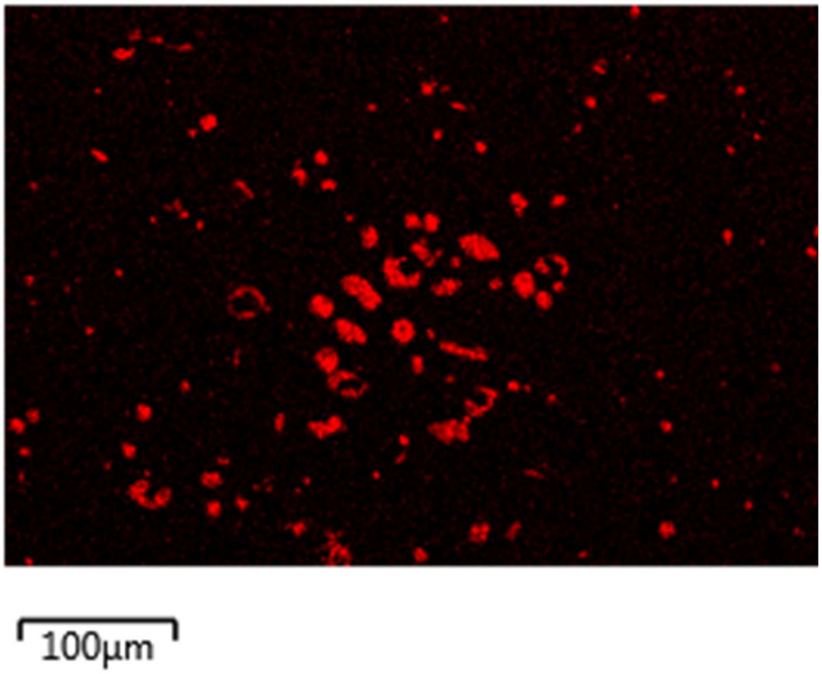

Figure S9. EDX analysis of PPy-Pt/SDS emulsion during CryoSEM. Red denotes the presence of carbon. In this case the hexadecane oil core. Image clearly shows a large number of non-spherical droplets.

Particles Formed During Dialysis

During dialysis the dialysis medium would begin to brown and increase in opacity. The dialysis medium was collected and analysed using Malvern ZetaSizer. The presence of particles and the colour change suggested the formation of PDA particles despite NMR measurements demonstrating almost no monomer remaining. It is thought that oligomers could continue to polymerise when placed in the dialysis medium. Due to the presence of the concentrated Tris-HCl buffer, the pH would allow for continued reaction. It is likely that the fresh water introduced the partially formed polymers to an additional excess of dissolved oxygen allowing for the polymerization to continue resulting in the formation of additional PDA particles.

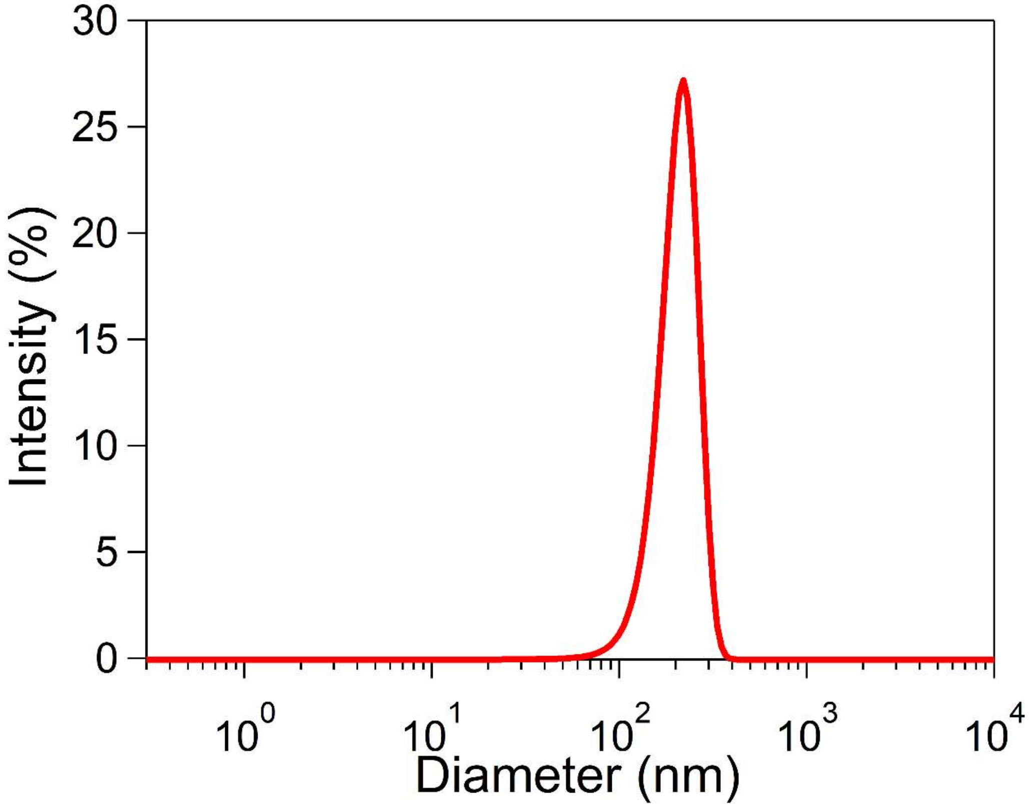

Figure S10. Size distribution of particles formed in PDA dialysis medium after 24 hours. Obtained from DLS measurements.

Interfacial Behaviour of PPy-Pt/SDS System

PPy-Pt/SDS particles were prepared as outlined in §2.1 of the main manuscript without the addition of hexadecane or sonication. Particles were then dialysed, and interfacial tension measured in the same manner as the PDA particles presented in the main manuscript. The exception to this was the preparation of the PPy particles without Pt-NPs embedded within their structure. These were prepared using $FeCl_3$ instead of $H_2PtCl_6.xH_2O$ to determine if the Pt particles played an important role in their interfacial behaviour. The pyrrole was oxidized by $FeCl_3$ in a 3:7 molar ratio at RT and allowed to stir for 24 h).[3-5]

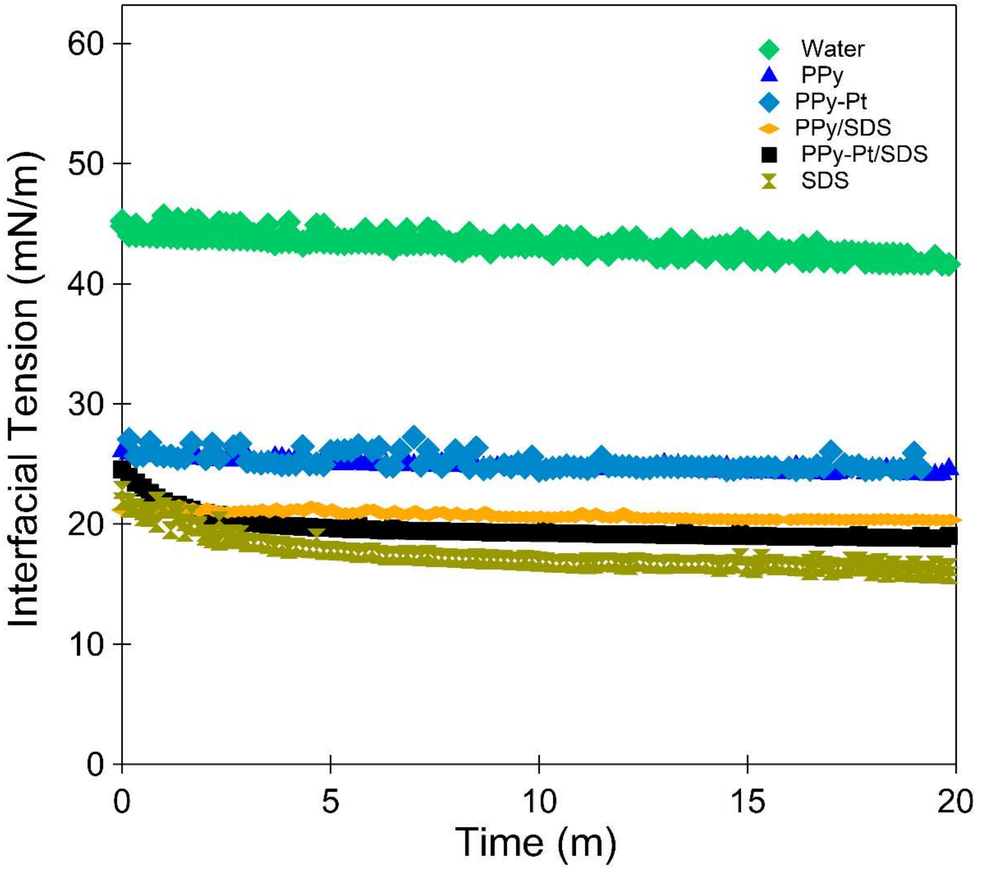

Figure S11. Interfacial tension measurement of PPy-Pt/SDS emulsion components against hexadecane oil.

Number and Volume Distribution of Emulsion Droplets as Determined by FlowCam

Larger droplets were not able to pass through the orifice of the flow cell and as such could not be imaged by the FlowCam. However, given the nature of the measurement (image analysis) the data that was able to be obtained does not suffer from the same limitations as laser diffraction. Specifically, the assumption of spherical droplets. Larger droplets observed in micrographs presented in the main manuscript for the 500 RPM sample cannot be analysed and as such the smaller droplets dominate in this sample for both number and volume distributions. FlowCam obtained size distributions of emulsion prepared at increasing shear rates using the overhead mixer are thus presented here.

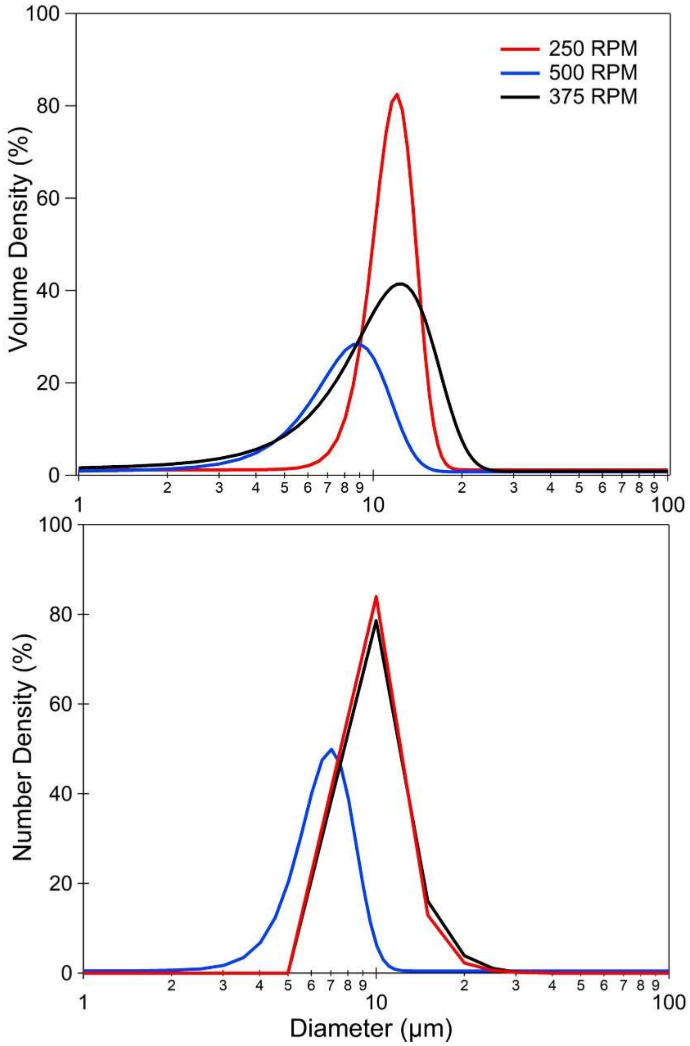

Figure S12. Size distributions of non-spherical emulsion droplets as determine by FlowCam analysis. Top: Volume Distribution, Bottom: Number Distribution.

Captions for Supporting Videos

Video S1 – Pendent droplet of suspension of polydopamine particles as prepared (PDA-AP) suspended in hexadecane oil phase, played at 21x speed, needle outer diameter 0.7 mm (22G).

Video S2 – Pendent droplet of suspension of dialysed polydopamine particles (PDA-D) suspended in hexadecane oil phase, played at 21x speed, needle outer diameter 0.7 mm (22G).

Video S3 – Pendent droplet of suspension of polydopamine particles post-dialysis and after the addition of CTAB and Tris at PDA-AP concentrations (PDA-CTAB-Tris) suspended in hexadecane oil phase, played at 21x speed, needle outer diameter 0.7 mm (22G).

References

1. H. Takeoka, H. Hamasaki, Y. Harada, Y. Nakamura, S. Fujii, Synthesis and characterization of polypyrrole-platinum nanocomposite-coated latex particles, Colloid and Polymer Science, 293 (2015) 1483-1493.
2. S.T. Knox, S. Parkinson, R. Stone, N.J. Warren, Benchtop flow-NMR for rapid online monitoring of RAFT and free radical polymerisation in batch and continuous reactors, Polymer Chemistry, 10 (2019) 4774-4778.
3. B.T. Lobel, J. Fujiwara, S. Fujii, C.A. Thomas, P.M. Ireland, E.J. Wanless, G.B. Webber, Formation of liquid marbles & aggregates: rolling and electrostatic formation using conductive hexagonal plates, Materials Advances, 1 (2020) 3302-3313.
4. S. Fujii, S. Matsuzawa, Y. Nakamura, A. Ohtaka, T. Teratani, K. Akamatsu, T. Tsuruoka, H. Nawafune, Synthesis and characterization of polypyrrole-palladium nanocomposite-coated latex particles and their use as a catalyst for Suzuki coupling reaction in aqueous media, Langmuir, 26 (2010) 6230-6239.
5. H. Kawashima, H. Mayama, Y. Nakamura, S. Fujii, Hydrophobic polypyrroles synthesized by aqueous chemical oxidative polymerization and their use as light-responsive liquid marble stabilizers, Polymer Chemistry, 8 (2017) 2609-2618.
